# The cerebrospinal fluid virome in people with HIV: links to neuroinflammation and cognition

**DOI:** 10.3389/fmicb.2025.1704392

**Published:** 2025-11-04

**Authors:** Mattia Trunfio, Rossana Scutari, Valeria Fox, Elisa Vuaran, Raha Maryam Dastgheyb, Vanessa Fini, Annarita Granaglia, Francesca Balbo, Dora Tortarolo, Stefano Bonora, Carlo Federico Perno, Giovanni Di Perri, Claudia Alteri, Andrea Calcagno

**Affiliations:** ^1^Unit of Infectious Diseases, Amedeo di Savoia Hospital, Department of Medical Sciences, University of Turin, Turin, Italy; ^2^HIV Neurobehavioral Research Program, Departments of Neurosciences and Psychiatry, University of California San Diego, San Diego, CA, United States; ^3^Division of Infectious Diseases and Global Health, Department of Medicine, University of California San Diego, San Diego, CA, United States; ^4^Multimodal Laboratory Research Unit, Bambino Gesù Children’s Hospital IRCCS, Rome, Italy; ^5^Department of Oncology and Hemato-Oncology, University of Milan, Milan, Italy; ^6^Department of Neurology, Johns Hopkins University School of Medicine, Baltimore, MD, United States; ^7^Department of Informatics, University of Turin, Turin, Italy; ^8^UniCamillus International Medical University, Rome, Italy; ^9^Microbiology and Virology Unit, IRCCS Fondazione Ca’ Granda Ospedale Maggiore Policlinico, Milan, Italy; ^10^Department of Translational Medicine, University of Eastern Piedmont, Novara, Italy; ^11^SCDO Malattie Infettive, AOU Maggiore della Carità, Novara, Italy

**Keywords:** brain microbiome, central nervous system, HIV-associated neurocognitive disorders, depression, cognitive functions, bacteriophages, eukaryotic viruses, prokaryotic viruses

## Abstract

**Introduction:**

Despite durable viral suppression, neuroinflammation and neurocognitive complications remain common yet poorly understood in people with HIV (PWH). HIV alters human viromes, and virome perturbations have been linked to neurocognitive issues in people without HIV. Recently characterized, the brain and cerebrospinal fluid (CSF) viromes represent a new avenue to understand brain and mental health in PWH.

**Methods:**

This cross-sectional study analyzed 85 CSF samples (74 from PWH on suppressive antiretroviral therapy, and 11 from controls without HIV, CWH) through shotgun metagenomics for DNA and RNA viruses. Taxonomic composition (reads and contigs), diversity, and relative abundance (RA) of prokaryotic (PV), human eukaryotic (hEV), and non-human eukaryotic viruses (nhEV) were evaluated in relation to HIV status, markers of neuroinflammation/neurodegeneration, cognitive functions, and depressive symptoms. Sensitivity analyses and post-hoc cluster analysis on the RA of hEV, non-human viruses (NHV) and blood–brain barrier permeability were performed. Multivariable models assessed the relationship between cognition and clusters.

**Results:**

Of 46 read-positive CSF samples, 93.5% contained PV sequences, 47.8% hEV, and 45.6% nhEV. PWH displayed lower α diversity, although *p* > 0.05. At β diversity analysis, HIV status explained 3.4% of the variation in viral composition (*p* = 0.016). Contigs assembly yielded 13 samples positive for 8 hEV, 2 nhEV, and 6 PV. Higher RA of PV was correlated with higher CSF S100β (rho 0.36, *p* = 0.002) and β-Amyloid 1–42 fragment (βA-42, rho 0.27, *p* = 0.026), whereas higher RA of nhEV with poorer cognitive performance (rho 0.26, *p* = 0.022). Conversely, higher RA of hEV correlated with better cognition (rho −0.38, *p* = 0.003) and lower βA-42 (rho −0.30, *p* = 0.012). Sensitivity analyses restricted to only CSF samples with detectable reads confirmed these findings. Three CSF clusters were identified and showed differences in astrocytosis, βA-42, tau protein, and cognitive functions. Participants with hEV-enriched CSF showed better cognitive performance compared to those with virus-devoid and NHV-enriched CSF (all *p* < 0.05).

**Conclusion:**

This study provides the first comprehensive description of the CSF virome in PWH, revealing associations with neuroinflammation and cognition. These findings highlight the potential involvement of the CSF virome in brain health and inform about its composition, origin, and potential clinical implications in people with and without HIV.

## 1 Introduction

The recognition of the human virome is not recent, even for the “sterile” central nervous system (CNS) (Link, 2021). Viruses that establish latent chronic infections have long been recognized as permanent residents of the human body, and neurotropic viruses (e.g., *Herpesviridae*, *Retroviridae*) have been represented the CNS virome (Link, 2021). Thanks to next-generation sequencing, the human virome has expanded to include a broader range of non-pathogenic viruses persistently or transiently colonizing the body (Liang and Bushman, 2021). It is now recognized that the largest part of the human virome consists of commensal viruses, primarily bacteriophages (phages), that infect non-human cells (Rascovan et al., 2016; Moustafa et al., 2017; Liang and Bushman, 2021; Cao et al., 2022).

The human virome is not a passive passenger: viral communities across the body exert local and systemic effects by which they can also influence distant organs such as the CNS (Liang and Bushman, 2021; Popescu et al., 2021; Bai et al., 2022; Mayneris-Perxachs et al., 2022; Castells-Nobau et al., 2024). Phages can influence the host’s bacteriome, metabolism (Shkoporov et al., 2022), immune system (Neil and Cadwell, 2018; Popescu et al., 2021), and disease risks (Wahida et al., 2021; Kviatcovsky et al., 2023). The gut phages *Siphoviridae* and *Microviridae* affect cognition and behavior in humans, mice, and *Drosophila*, promoting memory-related gene expression (Mayneris-Perxachs et al., 2022). In mouse models of chronic stress, a *Microviridae*-enriched virome alleviates anxiety and restores stress-induced changes of circulating immune cells, cytokines, and gene expression in the amygdala (Ritz et al., 2024). *Chlorovirus* ATCV-1 in the human oropharynx has been linked to lower cognitive performance by altering synaptic plasticity and learning/memory-related genes in the hippocampus (Yolken et al., 2014).

While research is uncovering the effects of the human gut microbiome on brain and mental health, the contribution of the CNS virome to its host organ’s health is largely unknown. For long, few bacterial, parasitic, and viral infections have been the target of investigations of a microbial etiology of neuroinflammation (Davis et al., 2021; de Haan et al., 2021; Link, 2021; Bjornevik et al., 2023; Levine et al., 2023), and the comprehensive study of the human CNS virome has just begun. A rich variety of prokaryotic, non-human, and human eukaryotic viruses with and without pathogenic potential or tropism to the CNS have been detected in human brain and cerebrospinal fluid (CSF) in the last decade (Branton et al., 2013; Ghose et al., 2019; Blanco-Picazo et al., 2020; Ghorbani, 2023; Pyöriä et al., 2023; Dang et al., 2024). Yet, few studies assessed the role of these viral communities in mental health (Ghorbani, 2023).

HIV affects the human microbiome throughout all stages of infection (Villoslada-Blanco et al., 2022; Ortiz and Brenchley, 2024). HIV-associated changes in immune cell activation, senescence, and exhaustion lead to shifts in the microbial communities, including viruses, across the body (Monaco et al., 2016; Ishizaka et al., 2021; Monaco, 2022; Lippincott et al., 2024; Ortiz and Brenchley, 2024). In turn, such microbiome perturbations can contribute to further inflammation and risk of comorbidities (Bai et al., 2024; Bulnes and Utay, 2024; Lippincott et al., 2024; Ortiz and Brenchley, 2024).

HIV is also a neurotropic virus that can affect brain and mental health. Aging PWH face up to 58% greater risk of all-type dementia compared to age-matched people without HIV (Lam et al., 2021; Hyle et al., 2024). Prevalence of HIV-associated cognitive disorders is estimated around 40% (Trunfio et al., 2018; Wang et al., 2020), and depressive disorders are two to three times more common in PWH than in the general population (Du et al., 2023). Neuroinflammation plays a pivotal role in both cognitive and mood disorders, especially during suppressive ART (Mudra Rakshasa-Loots et al., 2022; Wojna, 2022), but the exact causes remain poorly understood.

While the gut bacteriome is increasingly implicated in neuropsychiatric comorbidities among PWH (Rich et al., 2020; Carrico et al., 2022; Hua et al., 2023; Chen et al., 2024), classical neurotropic viruses (e.g., CMV, EBV, HSV-1) have likewise been linked to neuroinflammation, neurodegeneration, and worse cognition in PWH (Letendre et al., 2018; Trunfio et al., 2022b, 2023b), and recent evidence further suggests that the brain virome may differ between people with and without HIV (Dang et al., 2024). Together, these observations provide a strong rationale to test whether the CNS virome is involved in brain health in PWH.

We investigated the presence and composition of the CSF virome in PWH. We hypothesized that the CSF virome of PWH differs from that of people without HIV. We expected that its characteristics are associated with factors that are both site-specific (e.g., blood–brain barrier-BBB-permeability) and common to other human body viromes (e.g., CD4+ T cell count). We further expected that these differences would be associated with different levels of neuroinflammation, cognitive performance, and depressive symptoms. To test these hypotheses, we performed shotgun metagenomics for DNA and RNA viruses in CSF samples from PWH and participants without HIV infection (controls, CWH) and assessed the virome in relation to HIV status, CSF markers of neuroinflammation/neurodegeneration, and neuropsychiatric assessment. The virome was described using both an assembly-free (reads) and an assembly-based taxonomic annotation (contigs) after two considerations: (1) short viral genomic fragments from degraded virions can trigger immune responses (e.g., through DNA/RNA sensing) (Cai et al., 2021; Champagne-Jorgensen et al., 2023); these short, immunologically active sequences can be discarded by contig assembly, underestimating the quantification of biological players (Ayling et al., 2020); (2) The choice of one method over the other should be based on the characteristics of the target virome (e.g., expected richness, occurrence of rare species, average reads length and abundance), and very limited data are available on the CSF virome to tailor a pipeline on established references. Through this hypothesis-generating study we compared these two approaches to provide further information to future studies in the field.

## 2 Materials and methods

### 2.1 Study design and population

We performed a cross-sectional study in 85 CSF samples from 74 adult PWH and 11 adults CWH to describe the presence and composition of the CSF virome and investigate its relationships with HIV infection, neuroinflammation, depressive symptoms, and cognitive performance.

CSF samples from PWH were collected and stored between 2010 and 2019 at the Unit of Infectious Diseases, Amedeo di Savoia hospital, Torino (Italy) and retrospectively analyzed for CSF virome through shotgun metagenomics. From the CSF library, CSF samples of ≥1 mL were selected based on the following: (A) Inclusion criteria: age ≥18 years, plasma HIV-RNA < 200 cp/mL, being on standard three-drug 2NRTI-based therapy since at least 12 months, and signed consent for the storage and use of samples and data for future analyses at the time of lumbar puncture; (B) Exclusion criteria: diagnosis of major confounding factors for neurocognitive evaluation and CSF biomarkers prior or at CSF collection (e.g., CNS disorders or infections, language barriers, cerebrovascular events, head trauma, neuropathy), and substance or alcohol use disorders in the previous year. Among eligible samples, priority was given to those with available CSF biomarkers (measured at the same time of CSF collection), neuropsychiatric assessment (conducted within ±1 month of CSF collection), and female representativeness.

The 11 CWH were enrolled between March and June 2023. HIV-1/2 infection was ruled out at the time of CSF collection through fourth-generation commercial serology assay. Participation was prospectively offered by the Unit of Emergency Medicine, Reanimation and Anesthesia at Maria Vittoria hospital, Torino (Italy), to adult individuals undergoing spinal anesthesia for surgical indications, and negative medical history for immunological and CNS disorders (e.g., acquired or congenital immune deficits, cancer, immuno-rheumatological diseases, neurodegenerative disorders) and active infections. All CWH signed an informed consent. Spinal anesthesia was required for orthopedic (femur fractures, coxarthrosis, meniscus injury), urology (transurethral prostate resection, varicocelectomy), and abdominal surgery (hernioplasty, hemorrhoids, polyps’ removal).

The research was performed in accordance with the Declaration of Helsinki and has been approved by the Comitato Etico Interaziendale A.O.U. Città della Salute e della Scienza di Torino, A.O. Ordine Mauriziano di Torino, A.S.L. Città di Torino (protocol n.285/2022).

### 2.2 Clinical assessment and CSF biomarkers

Standardized assessments and physical examination were performed at the time of CSF collection to record demographics, clinical, HIV disease characteristics, and biomarker data.

CSF samples for metagenomics were stored at −80 °C within 1 h from the lumbar puncture. Paired CSF samples were immediately analyzed for: standard biochemistry (cells, glucose, and protein); markers of neurodegeneration (total tau, 181-phosphorylated tau [ptau], and fragment 1–42 of β amyloid [βA-42]; commercial immunoassays, Innogenetics, Ghent, Belgium, EU); markers of neuroinflammation (neopterin and S100β protein; commercial immunoassays, DRG Diagnostics, Marburg, Germany, and DIAMETRA Srl, Spello, Italy); BBB integrity through the CSF-to-serum albumin ratio (CSAR, CSF albumin mg/L:serum albumin mg/L); intrathecal synthesis by IgG index (CSF IgG:serum IgG:CSAR) according to age-adjusted Reibergrams [133].

Cut-offs for normality of the CSF biomarkers were based on standardized (e.g., CSF cells > 5/mL; CSAR <6.5 in subjects aged <40 years and <8 in older subjects) and manufacturer cut-offs for the general population, previously used in PWH (Motta et al., 2017; Caligaris et al., 2021; Trunfio et al., 2022a): tau >300 pg/mL (age ≤50 years), >450 pg/mL (age 51–70 years), >500 pg/mL (age > 70 years); ptau >61 pg/mL; βA-42 < 500 pg/mL; neopterin >1.5 ng/mL; S100β > 380 pg/mL.

HIV RNA was quantified in plasma and CSF by reverse-transcription PCR (Roche Amplicor, lower limit of quantitation 20 copies/mL). Results of PCR for EBV DNA, CMV DNA, and JCV DNA (in-house standardized real-time PCR) were available in the CSF for a subsample of PWH (Supplementary Table S2).

### 2.3 Mood and cognitive assessment

A subgroup of PWH (*n* = 61) underwent a comprehensive battery of cognitive tests within 1 month from CSF collection. Raw scores were converted to demographically corrected T scores by referencing to published normative standards which correct for age, education, race, and sex. Five cognitive domains were assessed by 13 tests (Supplementary Table S8): attention/working memory, executive functions, short- and long-term memory (auditory and visual), verbal fluency/language, and motor functions. Individual domain scores were combined to calculate the Global Deficit Score (GDS), as previously described (Blackstone et al., 2012); a GDS ≥ 0.5 indicated neurocognitive impairment (Blackstone et al., 2012).

Depressive mood was assessed in a subgroup of PWH (*n* = 66) through the Beck Depression Inventory II (BDI-II) (Beck et al., 1996). Cut-off for the severity of depressive symptoms have been validated in the general population, and are commonly used in PWH (Hobkirk et al., 2015; Petersen et al., 2023): a total score ≤13 is considered absent/subthreshold depression, 14–19 mild, 20–28 moderate, and 29–63 severe depressive symptoms.

### 2.4 Shotgun metagenomics

#### 2.4.1 CSF samples preparation and nucleic acid extraction

Prokaryotic and eukaryotic viruses in CSF were first enriched by isolation and purification of virus-like particles. This purification was performed through a filtration process that takes advantage of the smaller dimension of viruses with respect to eukaryotic and prokaryotic cells. CSF samples were initially filtered with 0.45 μm polyethersulfone filters (Merck millipore) to retain most of the bacteria and cellular contaminants and then with 0.22 μm polyethersulfone filters (Merck millipore) capable of enriching viruses.

The resulting filtrate (~200–400 μL of sample) was used for nucleic acid extraction using the QIAamp MinElute Virus Spin Kit (Qiagen, Hilden, Germany), according to the manufacturer’s instructions. Both the concentration and the quality of the extracted nucleic acids were assessed by Nanodrop.

#### 2.4.2 Amplification and sequencing

One hundred ng input of purified nucleic acids was used for genome amplification and sequencing following a modification of the QIAseq® Single Cell RNA Library Kits with Unique Dual Indexes protocol. Briefly, a first reverse transcription, amplification and cDNA production were performed from total viral nucleic acids (RNA plus DNA) to obtain a pool of cDNA. The cDNA obtained from the previous step was first subjected to enzymatic fragmentation and then used for libraries preparation by using the QIAseq Single Cell RNA Amplified cDNA. The produced libraries were purified with QIAseq Beads, were validated using the High Sensitivity D1000 ScreenTape system on a Bioanalyzer (Agilent Technologies) and then quantified using a High Sensitivity Double Stranded DNA kit on a Qubit Fluorometer (Thermo Fisher Scientific). Normalized indexed DNA libraries were then loaded onto an Illumina High Output Flow cell cartridge v.2.5, and sequenced using the NextSeq 550 instrument (Illumina, San Diego, CA, USA) with 2 × 150-bp paired-end reads.

Three different runs for the CSF samples from PWH, and a fourth run for the CSF samples from CWH were performed. In each run, the blank samples were processed in parallel with CSF samples to document contamination background.

#### 2.4.3 Virome characterization

Demultiplexed raw reads were trimmed for adapter and quality (Phred score >28) and deduplicated using Fastp (v0.23.2) (Chen et al., 2018). The final read quality was assessed by FastQC (v0.11.9) and MultiQC (v1.12) (Ewels et al., 2016; Babraham Bioinformatics - FastQC A Quality Control tool for High Throughput Sequence Data, n.d.). All reads mapping to human genome (GRCh38) were removed from all the samples using bbsplit (BBTools). The remaining clean reads were transformed back to paired end reads using reformat (BBTools). Taxonomy was assigned using Kraken 2 (v2.1.2) (Wood et al., 2019) and visualized by Krona (v2.8.1) (Ondov et al., 2011), while Bracken (v2.7) (Lu et al., 2017) was used to estimate family abundance.

The sequencing results underwent two parallel methods of taxonomic assignment: based on reads and based on contigs reconstruction. Reads classified as viral (taxonID 10,239) were retrieved using the ‘extract_kraken_reads.py’ script of the KrakenTools suite v1.2. Taxonomy assignment was obtained by supporting each viral taxon with at least two unique reads and a Kraken confidence score >0.50, as suggested (Madison et al., 2023; Wright et al., 2023). Species observed from the blanks were removed from all samples (for details see Supplementary Table S9).

For contigs reconstruction, reads retained after the previous steps were *de-novo* assembled using metaSPAdes (v3.14.1) (Nurk et al., 2017) in paired-read mode with default settings. The obtained contigs were filtered for length ≥300 bp, as suggested (Ansari et al., 2020), using reformat from BBTools, and then queried against a nucleotide viral sequences collection retrieved from the National Center for Biotechnology Information (NCBI) Reference Sequence Database (RefSeq, 8,416 sequences) using Nucleotide BLAST (blastn) with an e-value of at least 1 × 10^−10^ and a percentage identity of at least 70%. Species observed by the contigs assembled from the blanks (without length filtering) were removed from all samples (for details see Supplementary Table S9).

Eukaryotic and non-eukaryotic viral species and their genome coverage were visualized through a heatmap constructed using the ggplot2 (v3.3.6) and pheatmap (v1.0.12) R packages (Wickham, 2016). The filtered Bracken report output files were used to generate a BIOM-format table using the kraken-biom python package (v1.0.1). The biom-format package (v2.1.12) (McDonald et al., 2012) was then used to add sample metadata to the BIOM table, which was then loaded into R studio. The sequences used in this work have been deposited in GenBank (released date 01/31/25; BioProject ID PRJNA1176451).

### 2.5 Statistical analyses

Data were presented as median (interquartile range, IQR), mean (standard deviation, SD), and number (proportion, %), as appropriate. Non-normally distributed variables were log_10_-transformed to reduce skewness. Missing data accounted for a maximum of 5.4% of the observations across 11 variables for PWH (reported in Supplementary Table S10) and were addressed by excluding the affected participants from the corresponding analyses.

We described αdiversity using the number of observed taxa (richness of samples), Simpson index (sensitive to the dominance of species), and Shannon index (sensitive to both richness and evenness of species distribution). β diversity analysis was based on Bray Curtis dissimilarity (occurrence and abundance of taxa) and Jaccard distance (presence/absence of taxa) through Permutation Based Analysis of Variance (PERMANOVA) with Adonis and Principal Coordinate Analysis (PCoA). The following characteristics of CSF virome were also described and referred as viral metrics: total number of viral reads per CSF sample and relative abundance of viral categories (number of reads of each category divided by total number of viral reads per sample). Viral categories were human eukaryotic viruses (HEV), non-human eukaryotic viruses (nhEV), and prokaryotic viruses (bacteriophages, PV). Single viral families were not analyzed individually due to the limited number of samples positive for each family.

Kruskal-Wallis rank sum test, Mann–Whitney U test, Fisher’s exact test, Chi-squared test, and Pearson and Spearman’s rank correlations were used based on data distribution. Hierarchical clustering was performed to identify distinct groups based on the relative abundance of HEV, non-human viruses (PV + nhEV), and BBB; the distance matrix was computed using Euclidean distance (Ma and Zhu, 2013). Prior to clustering, input data were log-transformed to stabilize variance and improve comparability across variables. Hierarchical clustering was conducted using Ward’s minimum variance method (Ward.D2), which minimizes the total within-cluster variance at each step and optimizes cluster formation (Murtagh and Legendre, 2014). The resulting dendrogram was visualized to assess cluster structure, and the final number of clusters was selected based on visual inspection (Milligan and Cooper, 1985). Generalized linear models were used to confirm the associations between CSF clusters and cognitive performance adjusting for variables that significantly differed between the clusters.

Negative samples at sequencing could be interpreted as biological (meaningful information) or non-biological zeros (e.g., due to limits in sequencing depth, degradation of genomic material) (Jiang et al., 2022). All CSF samples were included in the analyses investigating quantitative relationships (e.g., correlations between viral metrics and cognitive scores or biomarkers), considering as closely related biological zeros and samples without viral sequences. However, taking into consideration the long conservation time of specimens and differential stability of DNA vs. RNA, we also performed sensitivity analyses in only the samples positive for viral sequences, to assess how observed associations may change. Analyses were performed through RStudio v4.3.3 (R Core Team 2024, Boston, MA, US) and STATA v18 (StataCorp, College Station, TX, US).

## 3 Results

### 3.1 Study population

Eighty-five CSF samples from 74 PWH and 11 CWH were analyzed. As detailed in Supplementary Table S1, PWH were predominantly white (93.2%), male (67.6%), of middle age, with a long history of HIV infection and past AIDS (62.2%). The routes of HIV transmission were equally represented. HIV RNA was detectable in the blood and CSF of 17.6% (range: 23–125 cp/mL) and 27.0% PWH (range: 20–133 cp/mL). Median CD4+ T cell count and CD4/CD8 ratio were 447 cells/μL and 0.7. CWH were also predominantly white, males, and mildly older than PWH (*p* = 0.074; Supplementary Table S1).

### 3.2 Taxonomic composition of the CSF virome and differences by HIV status

Sequencing returned a total of 1,487.78 million reads of mean length of 149 nucleotides and a median of 14.08 (9.87–21.81) million reads per subject. After quality filtering and removal of human-host reads, a total of 125.50 million non-human reads were retained (median of 1.15 [0.54–1.90] million per subject). After removing non-viral and blanks’ reads (detailed in Supplementary Table S8), 16,039 viral reads were retained, with a median of 44 (12–131) reads per subject. Eventually, taxonomy assignment (requiring at least two unique reads and a confidence score greater than 0.5) retained 14,046 total reads from 46 non-zero CSF samples (54.1%), with a median of 17 (6–88) reads per sample, while contigs assembly retained 3,530 total reads from 13 non-zero CSF samples (15.3%; median of 142 [59–354] reads per sample).

#### 3.2.1 CSF virome according to reads

Of the 46 positive samples for viral DNA or RNA, 38 were from PWH (51.3%) and 8 from CWH (72.7%). The higher prevalence of positive samples in CWH did not reach statistical significance (Fisher’s *p* = 0.214) even after adjusting by age that differed between the groups (aOR = 2.95 [0.68–12.76] for CWH vs. PWH, *p* = 0.147).

Prokaryotic viruses (PV) comprised 71.2% of all CSF viral reads and were detected in 93.5% of the positive samples (median 30 reads [5–86] per sample), as detailed in Table 1.

**Table 1:** Taxonomic composition of the CSF virome according to reads.

Viruses’ host	Viral family	Prevalence of CSF viruses				Relative abundance of CSF viruses	
		By family (%) (*n* = 85 vs. 46)		Overall (%) (*n* = 85 vs. 46)		By family (%) (*n* = 46)	Overall (%) (*n* = 46)
Human Eukaryotic cells	*Adenoviridae*	2.353	4.348	25.880	47.827	0.249	16.155
	*Flaviviridae*	1.176	2.174			0.0996	
	*Genomoviridae*	10.588	19.565			2.269	
	*Herpesviridae*	3.529	6.522			13.288	
	*Papillomaviridae*	5.882	10.870			0.107	
	*Parvoviridae*	1.176	2.174			0.0356	
	*Poxviridae*	1.176	2.174			0.107	
Prokaryotic cells	*Autographiviridae*	3.529	6.522	50.587	93.480	0.341	71.234
	*Drexlerviridae*	1.176	2.174			0.0356	
	*Inoviridae*	1.176	2.174			0.0498	
	*Microviridae*	3.529	6.522			0.199	
	*Myoviridae*	14.118	26.087			30.972	
	*Podoviridae*	5.882	10.870			10.144	
	*Rountreeviridae*	2.353	4.348			0.285	
	*Siphoviridae*	18.824	34.783			29.208	
Non-human Eukaryotic cells	*Amalgaviridae*	1.176	2.174	24.702	45.654	3.386	12.613
	*Bromoviridae*	1.176	2.174			0.0569	
	*Circoviridae*	3.529	6.522			0.299	
	*Iridoviridae*	2.353	4.348			0.0711	
	*Phycodnaviridae*	5.882	10.870			0.199	
	*Pithoviridae*	1.176	2.174			0.0213	
	*Retroviridae*	1.176	2.174			0.0711	
	*Sarthroviridae*	1.176	2.174			0.0854	
	*Sphaerolipoviridae*	1.176	2.174			0.413	
	*Virgaviridae*	5.882	10.870			8.010	

Prevalence of CSF viruses reports the number of CSF samples positive for each viral family over the total study samples (*n* = 85; extreme left) and virome-positive samples only (*n* = 46; left); the same data for the viral families pooled into viral groups (viruses of human eukaryotic cells, of prokaryotic cells, and of non-human eukaryotic cells) follow. Relative abundance of CSF viruses reports the number of reads belonging to each viral family (left) and to each viral group (right) over the total viral reads in CSF positive samples. CSF, cerebrospinal fluid.

Three families of the order *Caudovirales* (double-stranded DNA viruses), *Siphoviridae*, *Myoviridae,* and *Podoviridae* were the most abundant PV, being detected in 34.8, 26.1, and 10.9% of positive samples, and representing the 29.2, 31.0, and 10.1% of all the reads, respectively (Table 1).

Human eukaryotic viruses (hEV) made up 16.2% of the total reads and were detected in 47.8% of the positive samples (median 10 reads [4–33] per sample). Among these, *Genomoviridae* (single-stranded DNA) and *Papillomaviridae* (double-stranded DNA) were present in the 19.6 and 10.9% of virome-positive samples and accounted for the 2.3 and 0.11% of the total viral reads detected. *Herpesviridae* family (double-stranded DNA) followed by frequency (6.5%) but was the primary contributor of hEV reads (relative abundance, RA, 13.3%) (Table 1).

**Figure 1:**
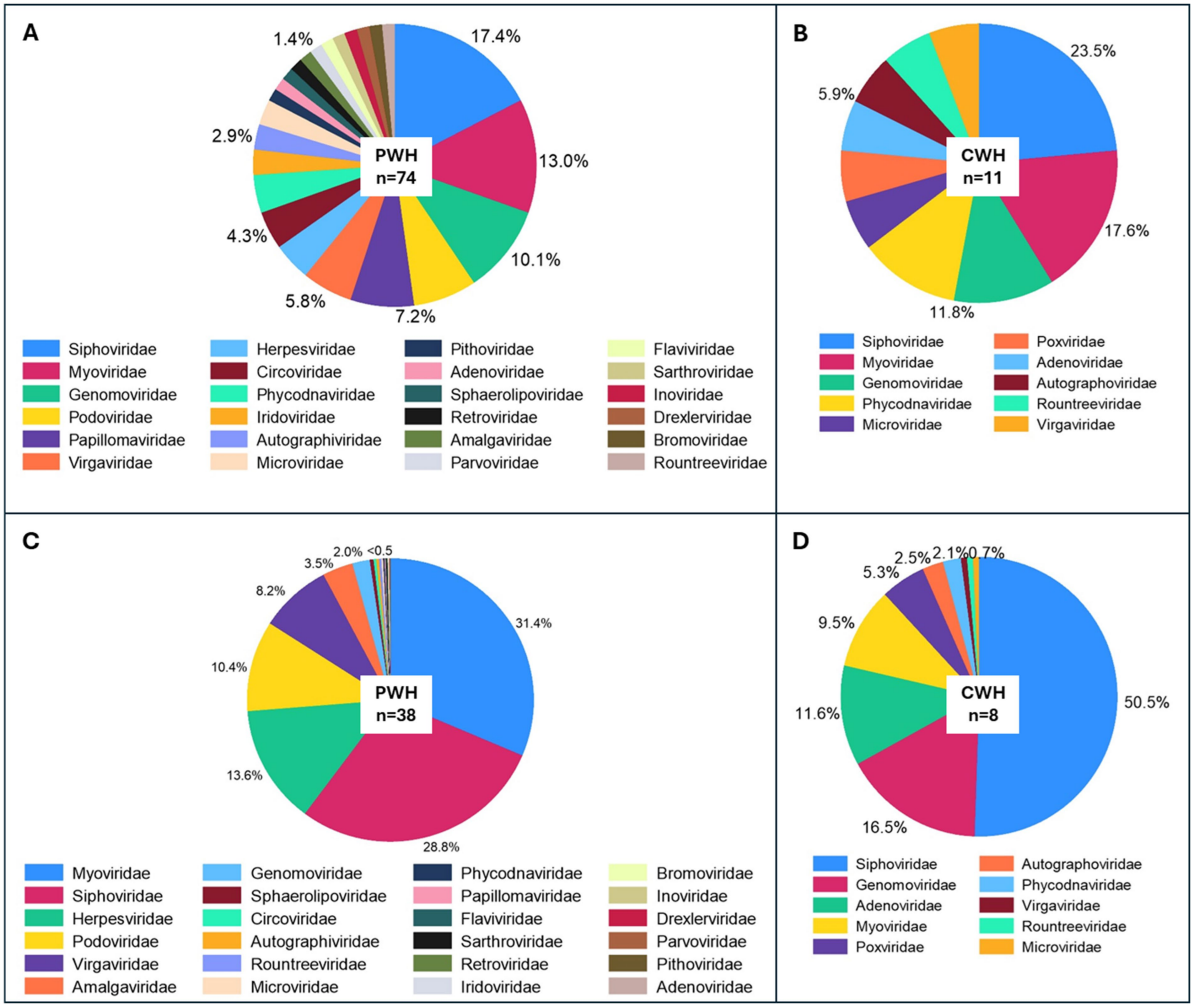
Prevalence and relative abundance of CSF viral families by HIV status. Panels **(A,B)** show the prevalence of viral families in the CSF (proportion of samples positive for each viral taxon among all the CSF samples) of PWH and CWH, respectively. Three families were found in about half of CSF samples from both PWH and CWH: *Siphoviridae* and *Myoviridae* (double-stranded DNA phages belonging to the order of *Caudovirales*), and *Genomoviridae* (single-stranded DNA viruses). Panels **(C,D)** show the relative abundance (number of reads of the specific taxon over the total number of reads per sample) of the viral families in virome-positive samples from PWH and CWH, respectively. *Myoviridae* and *Siphoviridae* represented the 60.2% of the total viral genomic material detected in the CSF of PWH, followed by *Herpesviridae* (13.6%, human double-stranded DNA viruses), *Podoviridae* (10.4%, double-stranded DNA phages belonging to the order of *Caudovirales*), *Virgaviridae* (single-stranded RNA viruses of plants), and 18 other viral families at much lower proportions. *Siphoviridae* contributed to the 50.5% of the total viral genomic material detected in the CSF of CWH, followed by *Genomoviridae* (16.5%), *Adenoviridae* (11.6%, human double-stranded DNA viruses), *Myoviridae* (9.5%), *Poxviridae* (5.3%, human double-stranded DNA viruses), and 5 other viral families at much lower proportions.

Non-human eukaryotic viruses (nhEV) constituted 12.6% of all CSF reads and were detected in 45.6% of the positive samples (median 4 reads [2–12] per sample). *Virgaviridae* (positive-strand RNA viruses of plants), *Phycodnaviridae* (double-stranded DNA viruses of algae)*, and Circoviridae* (single-stranded DNA viruses of animals) were the most frequent families. While *Virgaviridae* was also the main contributor of nhEV reads (8.0%), *Amalgaviridae* (double-stranded RNA viruses of plants and fungi) represented the second main contributor of nhEV reads (3.4%, Table 1). The composition of the CSF virome by families in PWH and CWH is shown in Figure 1.

**Figure 2:**
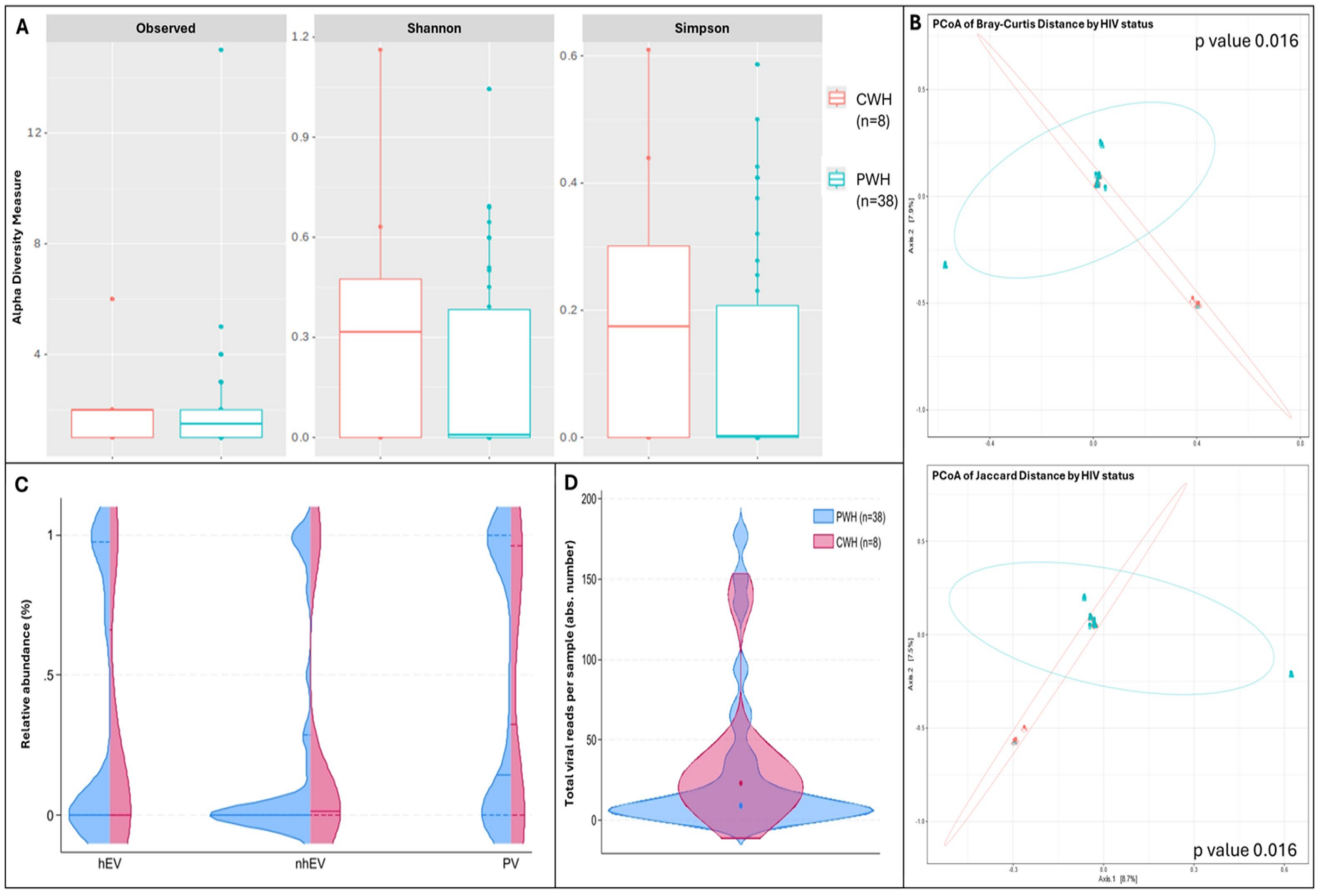
α and β diversity and CSF viral metrics by HIV status. Panel **(A)** shows the number of observed viral taxa (left), Shannon index (middle), and Simpson index (right) of the CSF virome against HIV status (CWH in orange, PWH in blue). While CSF samples from PWH had lower median values for each of the three metrics, none reached statistical significance. Panel **(B)** shows the representation of β diversity based on Bray Curtis (upper) and Jaccard distances (lower; CWH in green, *n* = 8; PWH in blue, *n* = 38; each dot represents a sample). A significant divergence in the composition of CSF viral communities based on HIV status was observed; Permutation Based Analysis of Variance (PERMANOVA) with Adonis function. Violin plots in panel **(C)** show the relative abundance of human eukaryotic viruses (hEV), non-human eukaryotic viruses (nhEV), and prokaryotic viruses (PV) reads in CSF samples from PWH (blue half; *n* = 38) and CWH (red half; *n* = 8); median and interquartile range are represented by continuous and dotted lines: hEV 0% (0–96.2) vs. 0% (0–49.1), nhEV 0% (0–25.6) vs. 1.4% (0–30.8), and PV 14.5% (0–100) vs. 32.5% (0–94.2) in PWH vs. CWH; when compared (Mann–Whitney U test), no significant difference was detected (*p* = 0.782 for hEV, *p* = 0.146 for nhEV, and *p* = 0.641 for PV). Panel **(D)** shows the violin plots (overlap) of the total number of viral reads detected in the CSF of PWH (median 15 reads per sample [6–124]; *n* = 38; blue violin) and of CWH (median 23 reads per sample [14–36]; *n* = 8; red violin); the median is represented by the dot; when compared by Mann–Whitney U test, no significance was detected (*p* = 0.247).

Compared to CWH, αdiversity was lower in PWH: the number of observed taxa was 1.5 [0.0–2.0] vs. 2.0 [1.0–2.0] and Shannon index was 0.00 [0–0.36] vs. 0.32 [0–0.47], suggesting lower richness and less even distribution among taxa in PWH; Simpson index was 0.00 [0–0.14] vs. 0.17 [0–0.30], confirming the dominance of fewer taxa in the CSF of PWH. Although all indexes were consistently lower in PWH, the differences did not reach statistical significance (*p* > 0.05 for all, *n* = 38 vs. *n* = 8; Figure 2A). β diversity analysis showed significant divergence in the composition of viral communities between PWH and CWH (*p* = 0.016 for both the distance measures; Figure 2B); however, HIV status explained only the 3.3 and 3.4% (R2 of Jaccard and Bray Curtis distance, respectively) of the variation in viral composition. When the RA of each viral family and of PV, hEV, nhEV were compared by HIV status, no difference was detected (Figure 2C).

Sequencing did not identify HIV-1 (including the 20 CSF samples with PCR-positive HIV RNA), EBV (in the 3 PCR-positive samples), and JCV (in both the PCR-positive samples). The detection limit of viruses in CSF through metagenomics, including HIV-1 RNA, has been previously described at ~10^2^ copies/mL (Bukowska-Ośko et al., 2016; Schlaberg et al., 2017; Miller et al., 2019); as the viral load of these viruses in all PCR-positive samples was <150 cp/mL (see Supplementary Table S3 for details), negative findings at sequencing were expected.

#### 3.2.2 CSF virome after contigs reconstruction

When only viral reads assembled into contigs of at least 300 nucleotides were considered, 10,516 reads had insufficient length, coverage, and overlap. After filtering these out, 13 CSF samples remained positive for viral contigs: 10 from PWH (13.5%) and 3 from CWH (27.3%). Accordingly, in PWH, the number of observed taxa was 1 [1–1], and both Shannon and Simpson index 0.0 [0.0–0.0]; in CWH, the number of observed taxa was 1, 1, and 4, Shannon index was 0.0, 0.0, and 0.92, and Simpson index was 0.0, 0.0, and 0.48.

**Figure 3:**
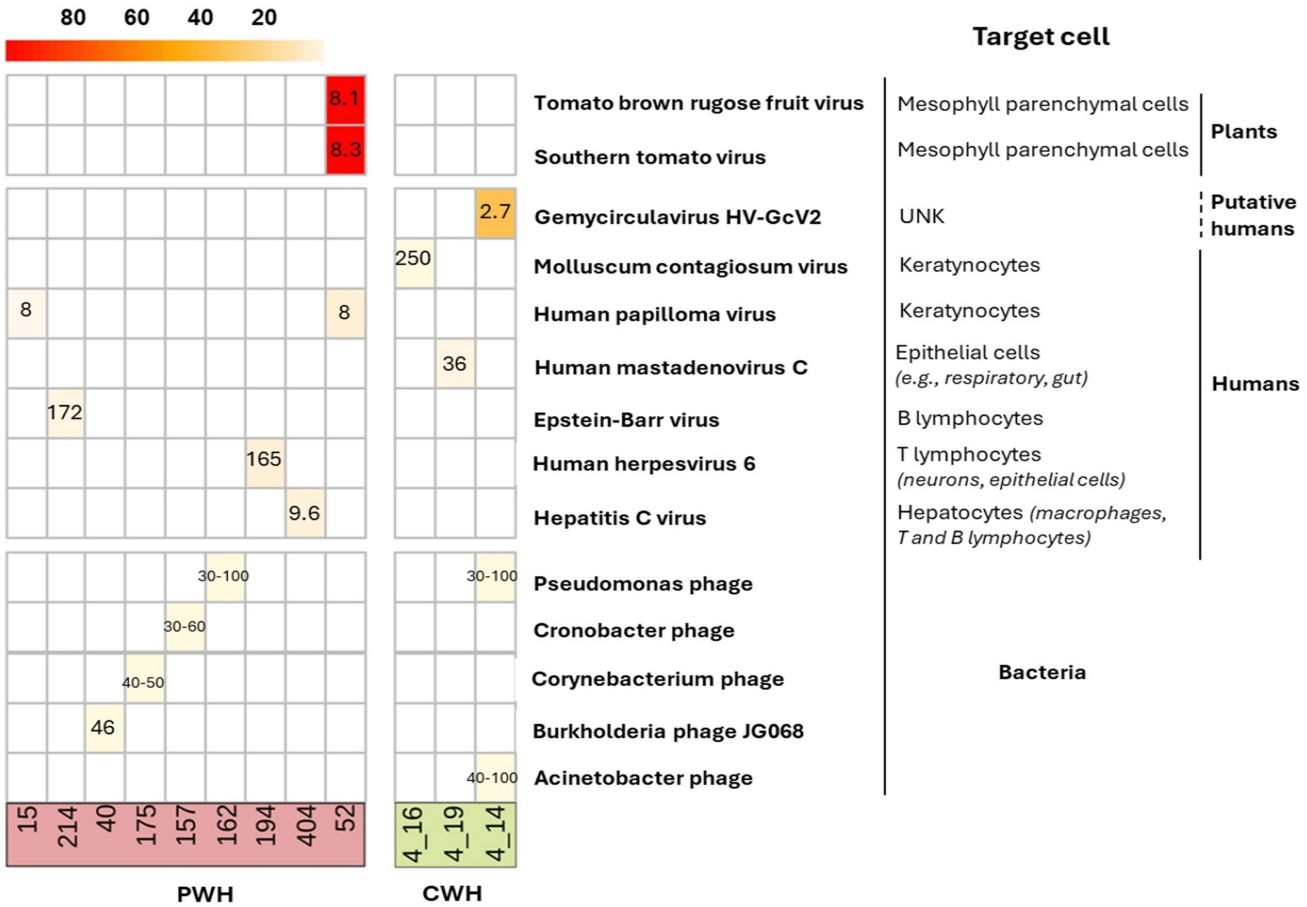
Heatmap of the genome coverage of CSF viruses detected after contig assembly. Each column corresponds to a CSF sample (PWH in red; CWH in green) and each row corresponds to the virus recovered. As shown in the heatmap, the genome coverage ranged from ~5% for the bacteriophages to almost the 100% for two non-human eukaryotic viruses, *Tomato brown rugose fruit virus* and *Southern tomato virus*, both detected in the CSF of one participant with HIV infection (ID52). The length of the genome of each identified CSF virus is reported in the corresponding box (range min-max for taxa with variability among species and strains). As detailed on the right, 13 out of 16 (76.5%) contigs belonged to viruses that have no target cells residing in or transiting through the CNS; EBV, HHV-6, and HCV have no primary target cells within the CNS. The mean and median length of CSF viral genomes were 914 (±3,859) and 2,835 (750–2,110) base pairs.

Contigs belonged to 8 unique hEV, 2 nhEV, and 6 unique PV (Figure 3). Among hEV, the dsDNA *Herpesviridae* family was represented by EBV and HHV-6 in one participant each, with 1,467 and 376 reads (genome coverage of 2.64 and 8.85%, respectively). HPV-12 and HPV-96 (in one participant each) were detected with 23 and 26 reads (genome coverage of 5.68 and 8.62%). The eukaryotic DNA viruses *Poxviridae Molluscum Contagiosum virus*, the *Human Mastadenovirus C* and the *Gemycircularvirus HV-GcV2* were also detected in one CWH each (27, 23 and 64 reads, genome coverage of 0.16, 1.78, and 33.7%). HCV was the only eukaryotic RNA virus identified and found in one sample from PWH with 98 reads and a genome coverage of 8.03%. The 2 nhEV were both viruses affecting tomato plants and other solanaceous crops, *Tomato Brown Rugose Fruit Virus* and *Southern Tomato Virus*, both found in one PWH, and presenting the highest genome coverage (98.3 and 94.6% respectively).

Of the 5 CSF samples positive for the 6 PV, all but one were from PWH (see Supplementary Table S5 and Supplementary material S1). None of these participants had positive medical history for infections with the bacterial hosts of the identified phages (e.g., *Acinetobacter* spp., *Pseudomonas* spp.). Of interest, the target hosts are all acknowledged commensal bacteria or opportunistic pathogens in the human gut, skin, and lung bacteriomes. The detailed characterization of these 12 participants is reported in Supplementary Tables S4, S5 and Supplementary material S1.

### 3.3 Exploratory analyses of other factors associated with the CSF virome

To assess whether other parameters were associated with the characteristics of the CSF virome, we performed exploratory analyses of the association between demographics and HIV-related parameters, α and β diversity, and viral metrics. Demographics and HIV-related parameters listed in Supplementary Table S1 (e.g., age, sex, CD4+ T cell count) were not associated with α diversity, viral metrics, nor with significant divergence of viral communities at β diversity analysis (data not shown). Due to the small number of positive samples, we did not perform these analyses after contig assembly.

#### 3.3.1 CSF virome and neuroinflammation

As secondary aim, we assessed whether α diversity and the RA of hEV, nhEV, and PV were associated with CSF biomarkers of neuroinflammation, neurodegeneration, and BBB permeability. We hypothesized that, regardless of the origin and size of the genomic material, its presence in the CSF can trigger immune activation and CNS injury. The CSF biomarkers are shown in Supplementary Table S6. The prevalence of BBB impairment was similar between PWH and CWH (21.6 and 18.2%), while intrathecal synthesis was detected only in PWH (27.0% vs. 0%, Supplementary Table S6). CSF-to-serum albumin ratio (CSAR), intrathecal synthesis, and CSF biochemistry (glucose, leukocytes, and proteins) were not correlated with diversity and viral metrics (data not shown).

As for the biomarkers of neuroinflammation/neurodegeneration, the CSF levels of βA-42 and S100β increased as the RA of PV increased (rho = 0.366, *p* = 0.002 and rho = 0.267, *p* = 0.026), while CSF βA-42 levels decreased as the RA of hEV increased (rho = −0.297, *p* = 0.012). At sensitivity analyses performed only in virome-positive samples (*n* = 38), the correlations between CSF βA-42 and RA of both PV and hEV were confirmed with larger effect size (rho = 0.633, *p* < 0.001 and rho = −0.435, *p* = 0.008).

β diversity analysis did not show divergence in the composition of the virome according to groups categorized by either median values of the biomarkers in the study population or standard clinical cut-offs (e.g., CSF proteins >45 mg/dL or CSF leukocytes >5 cells/mL; data not shown; the cut-offs used for each biomarker are detailed in the Methods). No differences in α diversity, total number of viral reads, or viral metrics were also observed when participants, all and then PWH only, were compared by BBB impairment and presence of intrathecal synthesis (data not shown).

In summary, weak-to-moderate associations were observed between the RA of CSF phages and hEV and CSF biomarkers of amyloid metabolism and astrocytosis. Contrary to our expectation, BBB impairment and intrathecal synthesis were not associated with higher CSF levels of viral material nor with distinct virome signatures.

#### 3.3.2 CSF virome, cognitive performance, and depressive symptoms

Then, we assessed whether CSF diversity and viral metrics were associated with cognitive performance and depressive symptoms. These analyses were performed in the subgroup of PWH with available data: 61 (82.4%) for cognition and 66 (89.2%) for depressive mood. Prevalence of cognitive impairment was 34.4%, and depressive mood was also common (28.8%) but mostly mild (Supplementary Table S6).

α diversity was not associated with Global Deficit Score (GDS), single domain deficit scores, nor depressive symptoms. Similarly, β diversity analysis showed no divergence in viral composition by depression (cut-off of BDI-II ≥ 14) and cognitive impairment (GDS ≥ 0.5). The amount of CSF viral reads was also not associated with cognitive scores and depressive symptoms (data not shown).

**Figure 4:**
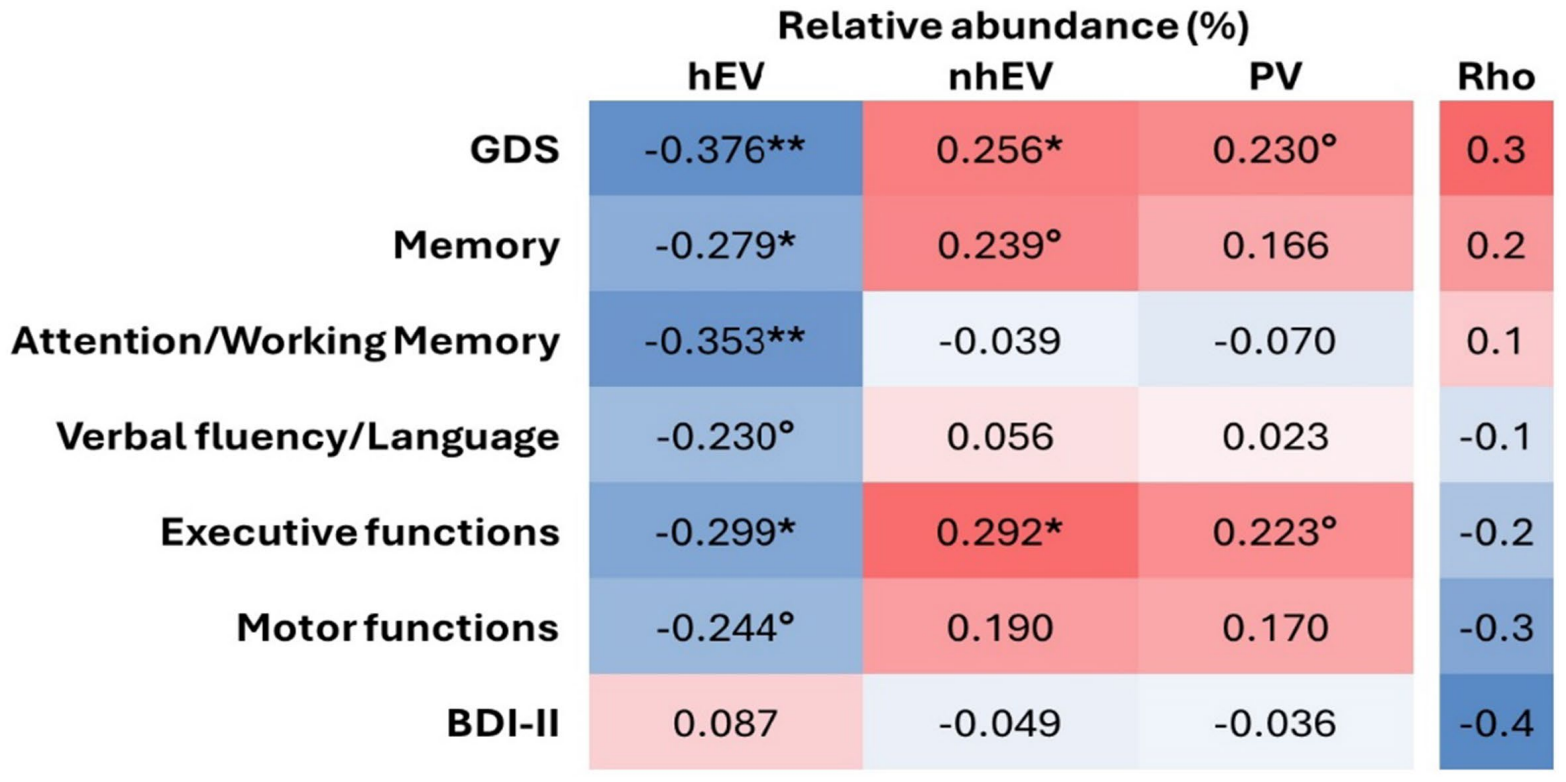
Heatmap of the correlations between relative abundance of CSF viral categories and neurocognitive measures. **p < 0.005; *p < 0.05; °*p* < 0.1 in *n* = 61 for cognitive metrics and *n* = 66 for BDI-II. hEV, human eukaryotic viruses; nhEV, non-human eukaryotic viruses; PV, prokaryotic viruses; GDS, Global Deficit Score; BDI-II, Beck Depression Inventory II.

On the contrary, higher RA of hEV was associated with lower GDS (better performance; Figure 4), and with better scores at memory, attention/working memory, and executive functions (a trend for better verbal and motor functions was also observed; Figure 4). Higher RA of nhEV was associated with worse global performance and worse executive functions (a trend for worse memory and motor functions was also observed; Figure 4). Higher RA of PV was borderline associated with worse global performance and executive functions. Depressive symptoms were not associated with viral metrics (Figure 4). Sensitivity analyses performed only in participants with virome-positive samples confirmed the same correlations (Supplementary Table S7).

In summary, diversity and amount of CSF viral sequences were not associated with cognition nor depression, whereas the relative abundance of PV, hEV, and nhEV was differentially associated with global and domain-specific cognitive performance.

### 3.4 *Post hoc* analysis: CSF virome clusters

**Figure 5:**
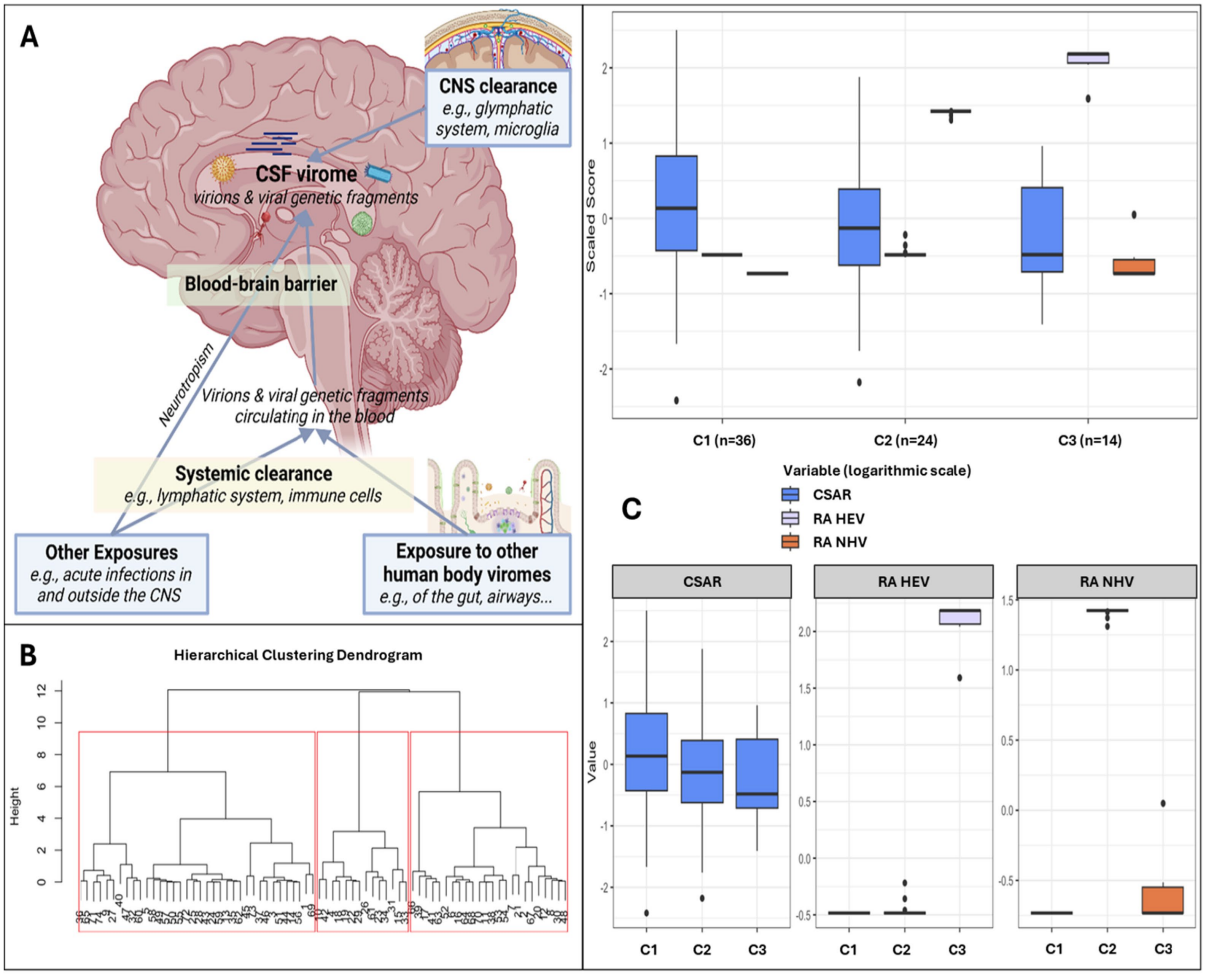
Clusters based on relative abundance of human and non-human CSF viruses and blood–brain barrier permeability. Panel **(A)** shows the hypothesis model: the human CSF virome is the collection of viral sequences that can come from both live virions and fragments of genomic material from death viruses. Part of the virome is generated within the CNS from viruses infecting resident or transiting cells (e.g., neurotropic viruses, viruses transported into the CNS by cells through trojan horse mechanisms), while part is represented by viral fragments that escape immune clearance and that originate from viruses that infect cells in blood and in other peripheral sites (e.g., gut, airways). These fragments eventually can enter the CNS as either free fragments (e.g., at the choroid plexus, through transcytosis, pinocytosis, or at leaking points along impaired BBB) or through trojan horse mechanisms (e.g., cells that phagocyted the fragment in the bloodstream and then migrated into the CNS). Therefore, the CSF virome (amount and type of viral sequences) will depend on a broad range of factors: e.g., spillover from the viromes of other body sites, ongoing viral infections, BBB permeability, peripheral immune clearance, and the clearance in the CNS operated by local cells and the glymphatic system. Within our study, the peripheral exposure, the systemic and the CNS clearance were unmeasured variables. Panel **(B)** shows the dendrogram for hierarchical clustering and the 3 clusters identified in red squares. Panel **(C)** shows the three clusters based on CSF-to-serum albumin ratio and relative abundance of human and non-human (prokaryotic and eukaryotic) viruses.

Having found a large proportion of CSF viral sequences belonging to viruses that do not infect CNS-resident cells, we hypothesized that most of these sequences originate from the periphery. Based on this hypothesis, illustrated in Figure 5A, we merged PV and nhEV reads into a single category, non-human viruses (NHV). This decision reflects their shared biological nature (genomic fragments derived from viruses in peripheral sites) and their potential indirect activity [e.g., triggering neuroinflammation through nucleic acid sensing and immune activation (Cai et al., 2021; Champagne-Jorgensen et al., 2023)]. In contrast, hEV reads were kept separate, as these viruses (e.g., Herpesviridae, Adenoviridae) included viruses previously shown to productively infect CNS cells. We then clustered participants by the relative abundance of hEV and NHV and by BBB permeability. While earlier analyses explored associations assuming linear relationships with demographics, cognition, and biomarkers, due to the lack of references to group participants by virome characteristics, this *post hoc* analysis examined the same relationships after grouping participants according to CSF viral metrics that showed biological signals. Clustering was performed only in PWH as we explored neurocognitive parameters. However, when CWH were included to assess the consistency of the clustering solutions, similar clustering results were observed (data not shown).

**Figure 6:**
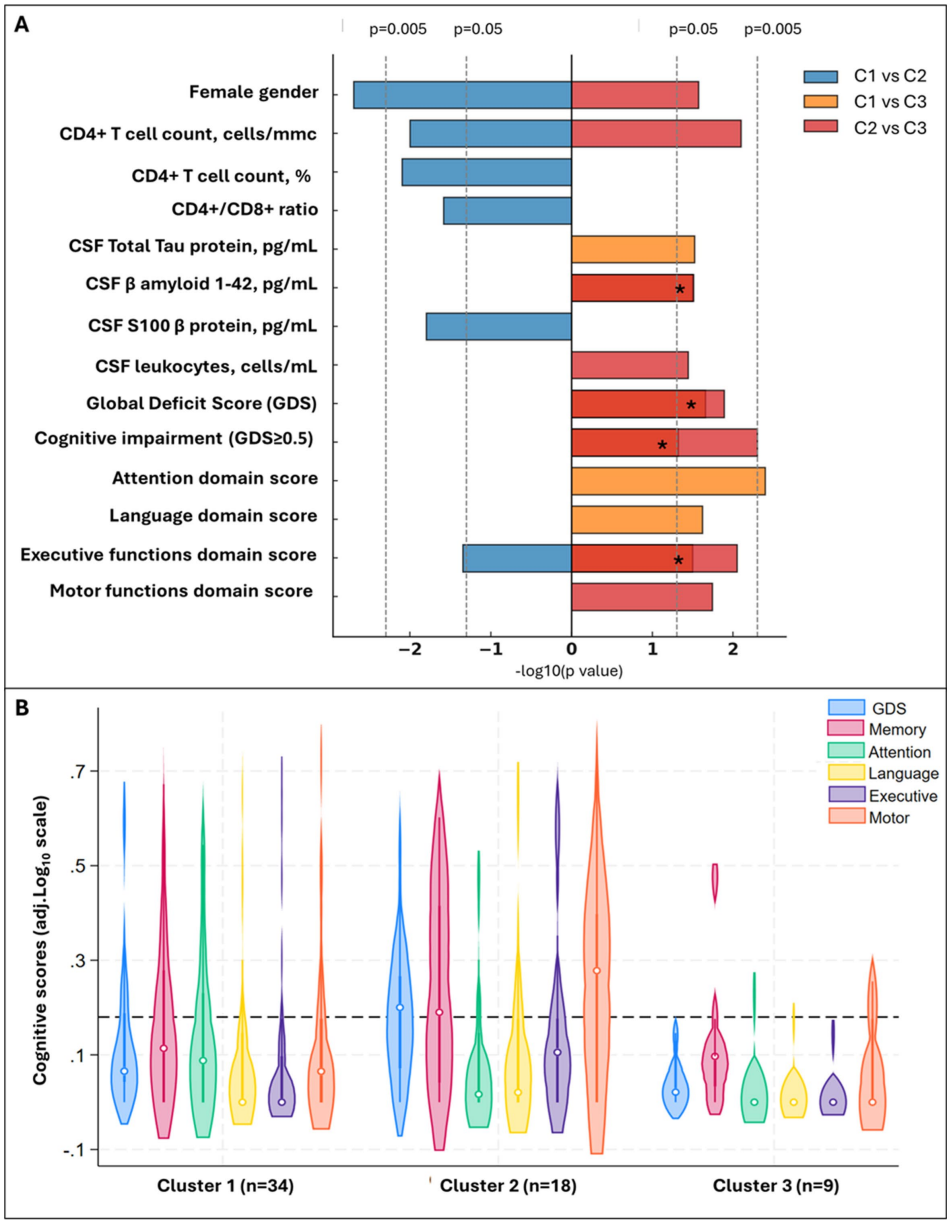
Significant differences between CSF clusters and Cognitive phenotypes of CSF clusters. Panel **(A)** shows demographic, CSF biomarkers and cognitive scores that significantly differ between clusters (pairwise comparisons with *p* < 0.05), with bar length proportional to −log10(*p*). Colors encode the comparison: blue = C1 vs. C2, orange = C1 vs. C3, red = C2 vs. C3. Bars extend right when the first cluster in the comparison has the higher value (e.g., C1 > C2 for blue) and left when the second cluster is higher. Four orange and red bars overlaid on the same side (C1 vs. C3 and C2 vs. C3 both significant in the same direction); for these, the segment appears deep red and is marked with an asterisk (*). Panel **(B)** shows violin plots for the Global and Domain Deficit Scores across the clusters; the dotted line cuts at 0.5, threshold to discriminate between impaired (above) and non-impaired cognitive performance (below).

Three clusters were identified (Figures 5B,C): C1 (*n* = 36) included participants with no viral reads in their CSF, despite the highest prevalence of BBB impairment (Figure 5C and Supplementary Table S8); compared to C1, C2 (*n* = 24) had similar degree of BBB impairment, and the CSF was enriched in NHV and poor of hEV; C3 (*n* = 14) had the lowest prevalence of BBB permeability, and the CSF was enriched in hEV and poor of NHV (Figure 5C). Figure 6 and Supplementary Table S8 show the comparisons of demographics, HIV-related parameters, biomarkers, and neurocognitive metrics between clusters. Among the differences, C2 had the highest prevalence of female sex, and levels of CD4+ T cells and astrocytosis (S100β protein). C3 had the lowest levels of all CSF biomarkers, with total tau, βA-42, and leukocytes reaching statistical significance. None of the members of C3 had cognitive impairment (versus 56% in C2, *p* = 0.005, and 32% in C1, *p* = 0.048), attaining the best scores in all cognitive domains (Figure 6; Supplementary Table S8). In multivariate analysis adjusted by demographics and clinical parameters that differed between clusters (Supplementary Table S9), C3 retained better global, executive, and motor performance compared to C2, and better attention/working memory compared to C1. C1 had better motor performance compared to C2 (Supplementary Table S9; all *p* < 0.05).

## 4 Discussion

This study provides the first comprehensive description of the CSF virome in PWH. Contrary to our hypothesis, HIV had a weak influence on the virome composition and richness in a population of people with viral suppression and normal CD4 T cell counts. However, only HIV status was associated to the composition of CSF viral communities, while no other demographic or HIV parameter was significantly linked to diversity and composition. We found associations between cognitive performance and relative abundance of CSF phages, human, and non-human eukaryotic viruses. Neuroinflammation and neurodegeneration may play a role in these relationships as we also found associations between the relative abundance of these viral categories and markers of astrocyte activation, tau protein, and β amyloid.

Approximately half of the CSF samples contained viral sequences. Of these, 93.5% contained PV sequences, and about half hEV or nhEV. Overall, 83.8% of the CSF reads were from viruses that do not infect human cells, primarily phages, with smaller contributions from plant, algae, and fungal viruses. Among the hEV, most belonged to *Herpesviridae*, though we also detected viruses not known to infect cells within or migrating through the CNS. After contig assembly, the number of positive samples and viral diversity decreased, but non-neurotropic viruses still predominated, as 87.5% of contigs were from viruses that primarily infect bacteria, epithelial cells, hepatocytes, or plants.

Participants with CSF phages had no clinical or CSF biochemistry evidence of bacteria in the CNS. Given the virulence of the bacterial hosts (e.g., *Pseudomonas, Cronobacter, Corynebacterium, Burkholderia* spp.), the undetected presence of such bacteria in the CSF is unlikely. As discussed in Supplementary material S1, all the identified phages target bacteria that can reside in the gut, skin, and lung microbiome. Thus, these findings may suggest that most of the CSF viromecould originate from periphery.

The length and weight of the contigs (median 2,835 bp, ~1,843 kDa) should rule out the free passage of these sequences across the BBB, as hydrophilic molecules >450 Da hardly cross intact BBB (Meng et al., 2021; Pardridge, 2022; Sun et al., 2023). The sequences were also too large for pinocytosis, transcytosis, or filtration by choroid plexus (Lalatsa and Butt, 2018; Wu et al., 2023). We hypothesize that trafficking cells that have been infected by or have phagocyted these virions/fragments outside the CNS (e.g., macrophages, T cells) largely contributed to the amount of the viral material in the CSF. This interpretation is in line with the well acknowledged Trojan horse mechanism (Santiago-Tirado and Doering, 2017) and further evidence, such as peptidoglycan detection in brain lesions infiltrated with blood-derived leukocytes in people suffering from multiple sclerosis (Schrijver et al., 2001). When considering reads, some sequences had length and weight small enough to potentially cross both intact and injured BBB. Therefore, the presence of some sequences could also be explained by virions/fragments that escape mucosal barriers and immunity from peripheral niches (e.g., gut, lung, skin) (Cao et al., 2022; Tiamani et al., 2022). Once in the bloodstream, these can be filtered by the immune system, and small escaping genomic fragments may break through the BBB. This parallel circuit is supported by previous findings, like the detection of small *Helicobacter pylori* components activating astroglia in brain tissues (Xie et al., 2023). Lastly, filamentous phages, like some *Siphoviridae* detected in our samples, can adhere to epithelial cells via receptors and actively cross barriers through transcytosis and micropinocytosis (Nguyen et al., 2017; Cao et al., 2022; Tiamani et al., 2022).

Our findings are consistent with those in the largest study of the CSF virome in people without HIV (Ghose et al., 2019). Ghose et al. analyzed 20 CSF samples from participants with various conditions (e.g., encephalitis, dementia, schizophrenia, lymphoproliferative cancers), and found that most sequences belonged to bacteriophages, despite microbiologically proven absence of bacteria (Ghose et al., 2019). The authors hypothesized that body sites devoid of bacteria, such as CSF, acquire their phageome primarily from the gut. As the CSF virome was not closely related to that found in the stools, they ultimately found less support for this hypothesis (Ghose et al., 2019). However, our findings, in a larger cohort with less confounding diagnoses, do support this hypothesis, considering that the stool microbiome does not accurately represent that in the gut (Ahn et al., 2023; Yan et al., 2023), and the composition of the virome of sterile body compartments, such as the CSF and bloodstream, could result from the contribution of the virome of the gut and other compartments of the body. As example, the three most abundant families that we and Ghose (Ghose et al., 2019) detected in the CSF (*Siphoviridae*, *Myoviridae*, *Podoviridae*) are common residents of the human gut, lungs, oral cavity, and skin (Liang and Bushman, 2021), as all other detected phages (see Supplementary material S1). Another example in support of this are the low-level sequences of plant viruses detected in CSF. These are most plausibly peripherally derived (e.g., diet-associated, gut-origin fragments) and trafficked via blood–CSF exchange or cells. This interpretation aligns with the participant presenting diverticulosis/diverticulitis who harbored two tomato viruses despite a CSAR not suggestive of BBB injury (as discussed in Supplementary material S1). Since plant and algae viruses are a potential signal of environmental contamination, we treat these observations cautiously and emphasize our negative-control–based filtering.

An association between the amount of CSF viral sequences and BBB permeability would have provided indirect evidence supporting this model, but neither CSAR nor the age-adjusted classification of BBB injury were associated with the type and number of viral sequences. In this regard, we could not adjust our analyses by other relevant factors such as the activity of the glymphatic system (Bohr et al., 2022) and local scavengers (e.g., astrocytes and microglia) (Waltl and Kalinke, 2022). Both could contribute to the removal of viral sequences from the CSF. Furthermore, we could not take into account the heterogeneity of the possible mechanisms of CNS entry into our analysis. Thus, the conclusion that the CSF virome is not affected by BBB integrity could be premature and requires further investigation. This is also why we included CSAR as one of the clustering variables.

A complementary explanation for the presence of CSF phages is that a brain bacteriome does exist (Link, 2021). Branton et al. detected sequences of both bacteria and phages in autopsy-derived cerebral white matter tissues of people with and without HIV (Branton et al., 2013). The existence of a brain bacteriota was further supported by positive *in situ* staining of peptidoglycan and controlled experiments of transplantations of the CNS tissues into mice, that proved the viability of the bacteria (Branton et al., 2013). The distinction between a “brain microbiota,” referring to living microorganisms, and a “brain microbiome” hypothesis, meant as the pool of genomic fragments, remains a key question (Link, 2021). As we did not perform analyses for virus viability/infectivity, our findings are compatible with both the hypotheses, which are non-mutually exclusive. Because we did not collect contemporaneous paired blood virome data, we could not assess CSF–blood compartmentalization. Longitudinal studies on larger samples from blood, CNS, and other body sites, including different experimental steps (e.g., nuclease protection assays, transmission electron microscopy) and strain-level analyses (e.g., phylogenomic) will be needed to establish whether a compartment-specific CSF virome exists, the nature of the CSF viral sequences (e.g., virion-to-fragment ratios), their transient fluctuations or stability over time, the relationship between the viromes of different body compartments, and whether such signatures track with cognitive outcomes.

We found that HIV infection explains a small part of the variation in CSF viral communities. However, we did not identify unique viral species or other metrics that clearly distinguish the virome of PWH from that of CWH. The limited sample size of positive CSF and small representation of taxa limited the power of this analysis. Larger samples are also required to assess whether, despite viral suppression, HIV infection can decrease the diversity of the CSF virome, as we observed lower *α* diversity in PWH, but the difference did not reach statistical significance. In PWH off ART, with AIDS, or low CD4+ T cell counts, an expansion of phages and other NHV has been described in the blood (Li et al., 2012, 2013, 2022; Liu et al., 2021). On the contrary, ART-mediated viral suppression and CD4+ restoration can shift gut and blood viromes toward composition close to that of people without HIV (Liu et al., 2021; Monaco, 2022; Villoslada-Blanco et al., 2022). This can suggest a true lack of association between αdiversity and HIV status due to the good viroimmunological profile of our study population and explain the weak effect size of HIV on CSF virome composition. Direct comparisons of our findings with prior studies are hindered by differences in anatomical sites, demographic and viro-immunological characteristics of the study populations, including sexual behaviors (Liu et al., 2021), and sequencing methods (Li et al., 2012, 2022; Monaco et al., 2016; Guo et al., 2021; Liu et al., 2021; Monaco, 2022; Villoslada-Blanco et al., 2022; Bhagchandani et al., 2024; Dang et al., 2024). For example, a richer virome has been described in postmortem prefrontal cortex tissues of PWH compared to CWH (Dang et al., 2024). The difference with our findings can be ascribed to differences between *in vivo* and postmortem samples, study population, sequencing pipelines, and the possibility that CSF and brain viromes may not be closely related despite anatomical proximity.

Our second hypothesis was that the presence of viral sequences in CSF contributes to neuroinflammation and affects the brain and mental health of PWH. The etiology behind the HIV-associated neuroinflammation and increased risk of cognitive and mood problems is multifactorial and not fully elucidated (Winston and Spudich, 2020; Mudra Rakshasa-Loots et al., 2022; Ellis et al., 2023), with direct contributions of HIV less impactful during ART (Spudich et al., 2019; Calcagno et al., 2021; Chan and Spudich, 2022; Trunfio et al., 2022a). For example, intrathecal synthesis of immunoglobulins is a common feature of HIV-associated cognitive impairment and primarily driven by responses to non-HIV antigens (Bonnan et al., 2015; Trunfio et al., 2023a; De Almeida et al., 2021). Consistent with this, we and others have shown that immune responses against common viruses (e.g., HSV-1, EBV, CMV) contribute to neuroinflammation and cognitive impairment in PWH (Letendre et al., 2018; Trunfio et al., 2022b, 2023b), suggesting that non-HIV virus-directed intrathecal immunity may be one pathway connecting CNS inflammation to cognitive outcomes, in line with evidence in the general population (Balin and Hudson, 2018; Readhead et al., 2018; Bjornevik et al., 2022; Khalesi et al., 2023; Grut et al., 2024; Hakami et al., 2024; Ma et al., 2024). To date, however, the role of CSF virome in neurocognitive problems has not been investigated. Contrary to our hypothesis, we found no associations between the total amount of CSF viral sequences and neuroinflammation, neurodegeneration, cognition, or depressive symptoms. Intrathecal synthesis was also not associated with any characteristics of the virome. As CSF immunoglobulins mostly originate in brain-resident cells and not CSF floating cells (Bonnan et al., 2015), this lack of association may reflect weak quantitative and qualitative overlap between CSF and brain viromes. While we measured common pathways of neuroinflammation and neurodegeneration, we may have missed others [e.g., short viral sequences can trigger IFNγ production (Rehwinkel and Gack, 2020; Yang and Li, 2020)], which has been previously linked to neurocognitive issues in PWH (Williams et al., 2021). Instead, we found that the RA of hEV, nhEV, and PV was associated with markers of neurodegeneration (βA-42, total tau), astrocyte activation, and cognitive functions. Higher RA of hEV was linked to better global and across-domain cognitive performance as well as lower levels of βA-42. In contrast, higher RA of nhEV correlated with worse global performance and executive functions, and higher RA of PV with higher levels of βA-42 and S100β. The increased activation of astrocytes, represented by increased S100β (Michetti et al., 2023), suggests astrocytic responses to PV. Accordingly, cluster analysis showed greater astrocytosis in participants with NHV-enriched CSF compared to those with virus-devoid CSF. The role of S100β and astrocytes in response to non-human viruses requires further validation, but is biologically plausible, considering astrocyte phagocytic activity (Lee and Chung, 2021; Li et al., 2025) and involvement in antiviral innate immune responses (Jorgačevski and Potokar, 2023). Our biomarker panel did not include canonical markers of microglial activation (e.g., CD86) or cytokine/chemokine panels (e.g., MCP-1, IFN-γ, IL-6). Accordingly, the present data primarily inform astrocytic activity (S100β), neurodegeneration (tau, p-tau, Aβ1-42), and broad immune activation/inflammation (neopterin, intrathecal synthesis, CSF leukocytes and proteins). Consistent with the CSF S100β and leukocyte signals, non-HIV viral fragments may activate resident scavenger cells (astrocytes, microglia, macrophages), that serve as HIV reservoirs (Lutgen et al., 2020; Tang et al., 2023), and may modulate the inflammatory milieu, leukocyte trafficking, and immune-cell clonality, thereby influencing HIV reservoir dynamics in the CNS and cognitive decline. Future analyses incorporating complementary cell-type–specific markers, immune pathways (e.g., IFN-mediated responses), and reservoir features (e.g., cell-associated HIV DNA/RNA in CSF leukocytes) are warranted to clarify links between the CSF virome, neuroinflammation, cognition, and HIV persistence. We acknowledge that these interpretations assume a causal direction from viromes to host responses; given the cross-sectional design, reverse or bidirectional relationships (e.g., host inflammation shaping the CSF virome) are also plausible.

Interactions between viruses and βA-42 have been previously described (White et al., 2014; Bourgade et al., 2016; Eimer et al., 2018). Longitudinal studies should assess the trajectory and outcomes of the relationship herein observed, as βA-42 levels can increase to contain viruses, but when chronically stimulated, they can reduce due to amyloid plaque deposition (White et al., 2014; Bourgade et al., 2016; Eimer et al., 2018). In our study, only 5 PWH had abnormally low βA-42 levels, as seen in Alzheimer’s dementia; therefore, the association of CSF βA-42 levels with the RA of hEV and PV should be interpreted within a non-pathological range and suggests that PV sequences in the CSF may be a stronger trigger for βA-42 production compared to hEV or nhEV. In cluster analyses, PWH with hEV-enriched CSF had better cognitive performance and lower βA-42 compared to participants with NHV-enriched or virus-devoid CSF. While this seems opposite to the evidence that chronic hEV infection can contribute to neurodegeneration and neuroinflammation (Balin and Hudson, 2018; Letendre et al., 2018; Readhead et al., 2018; Bjornevik et al., 2022; Trunfio et al., 2022b, 2023b; Khalesi et al., 2023; Grut et al., 2024; Hakami et al., 2024; Ma et al., 2024), the role of hEV has never been investigated alongside that of other virome components. The link between higher hEV RA and better cognition may reflect a relative shift toward a more physiologic CSF virome rather than direct neuroprotective effects of hEV. Considering the compositional structure of relative abundance data, hEV RA tracks inversely with NHV RA, our putative marker of “viral dysbiosis” (e.g., due to increased trafficking, impaired CSF clearance). We therefore interpret the hEV signal as a proxy of preserved CSF immune surveillance and clearance capacity. Using C3 as reference “expected” CSF virome, the absence of viral sequences in C1 may reflect robust immune responses that clear the CSF thoroughly, but come along with higher inflammation, neuronal injury (higher total tau), and thereby cognitive disturbance (worse scores in attention, language, and executive functions). On the contrary, both C2 and C3 had their CSF populated by hEV and NHV, but NHV predominance in C2 may indicate impaired CSF clearance or “viral dysbiosis,” for which normative references and contributors are unknown (Rascovan et al., 2016; Liang and Bushman, 2021). NHV should not persist in CSF, lacking strategies and viable hosts; thus, the presence of their nuclei acids may result from impaired mechanisms of local clearance (e.g., glymphatic system, phagocytosis of glial cells). Notably, dysfunction of both the glymphatic system and astroglial phagocytosis have been linked to cognitive impairment (Galloway et al., 2019; Lee and Chung, 2021; Tang et al., 2022; Wang et al., 2023; Li et al., 2025). Thus, the composition of CSF virome could be an epiphenomenon of the true mechanisms behind cognitive impairment rather than the cause.

C2 had also more CSF leukocytes than C3. An influx of specific blood leukocyte types (e.g., intermediate monocytes) has been linked to larger HIV reservoir in the CNS, neuroinflammation, and cognitive impairment (Veenhuis et al., 2021; Rubin et al., 2024). The reason for such influx in some but not all PWH is unknown. The higher RA of NHV and worse cognitive performance in C2 may be both due to an enhanced influx of blood leukocytes harboring viral sequences into the CNS, or vice versa the higher RA of NHV may be the driver of such increased afflux. Once again, the CSF virome could be the result of other processes that underly cognitive issues or could directly contribute to them. Further studies are needed to provide normative references for the human CSF virome, define CSF viral dysbiosis, and help disentangling associations from causality.

Among the limitations of the study, the cross-sectional design limits causal inferences and directionality in the associations; we cannot conclude whether the differences in CSF viral communities occur during or due to HIV, nor whether the virome composition actively contributes to cognitive performance and neuroinflammation or is a bystander of processes responsible of both. In this regard, the CWH group was limited in size, and comparisons with PWH were likely underpowered. Similarly, demographic and clinical variables, including age and CD4 + counts, were not associated with virome diversity or composition; this may reflect the cohort’s midlife-skewed age distribution with few participants at the extremes, the viro-immunologically well-controlled profile with limited variance, and limited power to detect small effects. The retrospective nature of the analysis may have affected the detection of genomic material, particularly RNA, due to the long-term preservation of some samples. Although we condensed our analyses to four runs to minimize potential batch effects, we cannot completely rule them out. The virus-like particle enrichment step was implemented to reduce genomic background interference and enhance viral recovery in low-biomass fluids, as previously suggested (O’Flaherty et al., 2018; Ghose et al., 2019; Dang et al., 2024). However, this step, combined with size-filtration and standard cDNA library preparation that does not employ targeted enrichment steps (e.g., circular DNA-enriching amplification), may under-capture low-abundance, latent/integrated, larger, and circular DNA viruses. For example, although *Anelloviridae* (circular ssDNA viruses) commonly predominate in blood of people with (Ma et al., 2025) and without HIV (Sabbaghian et al., 2024), we did not detect them in CSF. This could reflect true CSF–blood differences and/or workflow-related limitations. Sequencing depth was unable to detect low-level sequences of HIV, EBV, and JCV, identified by RT-PCR, as expected for viral loads <100 cp/mL (Bukowska-Ośko et al., 2016; Schlaberg et al., 2017; Miller et al., 2019). Furthermore, all samples were positive for viral reads prior to applying confidence thresholds; although this strengthens confidence in taxonomic attribution, it may exclude scarce or highly divergent taxa. Thus, CSF virome richness and composition are likely underestimated for certain taxa. While grouping CSF viruses by target hosts fits with our hypothesis model, it may oversimplify the heterogeneity across viruses. Lastly, CWH had no neurocognitive assessment limiting the possibility to include this group in the specific analyses.

Among the strengths, the unbiased approach of shotgun sequencing and the *post hoc* clustering analyses, the comprehensive assessment of cognition, mood, and biomarkers in the largest set of CSF samples used for shotgun viral metagenomics, the comparison of an assembly-free vs. assembly-based method, the ecological validity of our study population in representing modern PWH, and the inclusion of internal negative control specimens and CWH. The use of blank controls may have resulted in the exclusion of some taxa truly present, but it ensured the highest confidence in the absence of contamination during the experiments. The risk of pre-analytical contamination was also minimized by sterilizing the skin at the LP site, using the last collected CSF sample (~7 tubes) for sequencing, and confirming the absence of red blood cells in all samples. Lastly, the use of specimens from living participants, rather than postmortem samples, avoids inevitable contaminations occurring after death, and provides insights on *in vivo* phenomena not confounded by postmortem processes.

In conclusion, this study offers the first comprehensive description of the CSF virome of PWH on suppressive ART and valid CD4+ T cell count. HIV infection exerted only a modest influence on CSF virome composition, despite being the single factor associated with it. Most CSF sequences were from viruses that have no recognized host within the CNS, suggesting a peripheral source for most of them. Half belonged to bacteriophages commonly found in other human viromes, followed by equal proportions of human and non-human eukaryotic viruses. The relative abundance of these viral categories was associated with cognition in PWH, with higher abundance of non-human eukaryotic viruses associated with poorer cognitive performance. Distinct viral genomic landscapes were also linked to diverse signatures of neuroinflammation and neurodegeneration, with higher abundance of phages associated with astrocytosis and higher abundance of human eukaryotic viruses associated with lower amyloid β. Further investigation is warranted to understand the mechanisms driving these relationships, identify the origin of the CSF virome, and explore its role in brain and mental health.

## Data Availability

The datasets presented in this study can be found in online repositories. The names of the repository/repositories and accession number(s) can be found here: https://www.ncbi.nlm.nih.gov/genbank/, PRJNA1176451.
