# Supplementary material for: The cerebrospinal fluid virome in people with HIV: links to neuroinflammation and cognition
Source: Front Microbiol. 2025 Nov 4;16:1704392. doi: 10.3389/fmicb.2025.1704392 (PMC12623318; doi:10.3389/fmicb.2025.1704392)
Supplement: Supplementary file 1 [file Data_Sheet_1.docx]

**Supplementary Material**

**S1 Table. Demographic and HIV-related characteristics**

| **Variable** | **N= 74 PWH** | **N=11 CWH** | **p-value** |
| --- | --- | --- | --- |
| **Age, years** | 49.6 (±11.4) | 57.0 (±19.5) | 0.074 |
| **Male sex, n** | 50 (67.6%) | 10 (90.9%) | 0.163 |
| **White race, n** | 69 (93.2%) | 11 (100%) | 1.000 |
| **HIV transmission routes, n**  **MSM**  **Heterosexual**  **pIDU** | 24 (32.4%)  26 (35.1%)  24 (32.4%) | - | - |
| **Plasma HIV RNA<20 cp/mL, n**  **Plasma HIV RNA, cp/mL°** | 61 (82.4%)  54 (33-100) | - | - |
| **CSF HIV RNA <20 cp/mL, n**  **CSF HIV RNA, cp/mL°** | 54 (72.9%)  48 (28-72) | - | - |
| **Current CD4+ T cell count, cells/µL** | 447 (318-677) | - | - |
| **Current CD4+ T cell count, %** | 28 (19-34) | - | - |
| **Current CD4/CD8 ratio** | 0.7 (0.4-1.0) | - | - |
| **CD4+ T cell count nadir, cells/µL** | 153 (47-260) | - | - |
| **Previous AIDS event, n** | 46 (62.2%) | - | - |
| **Estimated duration of HIV infection, years** | 11 (2-19) | - | - |
| **ART regimen, n**  **PI-2NRTIs**  **NNRTI-2NRTIs**  **INSTI-2NRTIs** | 31 (41.9%)  20 (27.0%)  23 (31.1%) | - | - |

Data are expressed as mean ± standard deviation, median (Interquartile range, IQR), or N (%). °Among participants with plasma/CSF HIV RNA >lower limit of quantification of 20 cp/mL; Legend: PWH, people living with HIV; CWH, control participants without HIV; MSM, males who have sex with other males; pIDU, past intravenous drug users; CSF, cerebrospinal fluid; ART, antiretroviral therapy; PI, protease inhibitors; NNRTI, non-nucleoside reverse transcriptase inhibitors; INSTI, integrase strand-transfer inhibitors.

**S2 Table. Viruses detected in the blank samples and removed from the study samples**

| **Blank** | **Virus Family** | **N reads** | **N contigs** | **Contig length (bp)** | **BLAST** | **BLAST Identity (%)** |
| --- | --- | --- | --- | --- | --- | --- |
| 1 | *Coronaviridae* | 14 | 3 | 68 (55-94) | *Severe acute respiratory syndrome coronavirus* | 100 (100-100) |
|  | *Retroviridae* | 19 | 8 | 192(146-220) | *Equine infectious anemia virus** | 100 (98.6-100) |
| ‍2 | *Suoliviridae* | 56 | 0 | - | *-* | - |
|  | *Retroviridae* | 7 | 1 | 574 | *Equine infectious anemia virus** | 100 |
| 3 | *Retroviridae* | 547 | 24 | 112 (106-146) | *Equine infectious anemia virus** | 100 (99.6-100) |
| 4 | *Retroviridae* | 33 | 2 | 235,223 | *Equine infectious anemia virus** | 98.8-100 |
|  | *Siphoviridae* | 20 | 1 | 219 | *Lactococcus phage* | 99.1 |
| 5 | *Circoviridae* | 993 | 1 | 1919 | *Cyclovirus* | 95,2 |
|  | *Retroviridae* | 148 | 17 | 123 (110-193) | *Equine infectious anemia virus** | 100 (100-100) |
| 6 | *Retroviridae* | 408 | 26 | 110 (104-143) | *Equine infectious anemia virus** | 100 (100-100) |
| 7 | *Retroviridae* | 70 | 5 | 164 (110-235) | *Equine infectious anemia virus** | 100 (100-100) |
|  | *Siphoviridae* | 79 | 5 | 279 (29-282) | *Propionibacterium phage Cutibacterium phage* | 94.1 (92.2-96.9) |
| 8 | *Retroviridae* | 191 | 15 | 116 (107-136) | *Equine infectious anemia virus** | 100 (98.9-100) |
|  | *Siphoviridae* | 98 | 3 | 219 (175-257) | *Propionibacterium phage Cutibacterium phage* | 97.8 (90.0-99.1) |

Contig length and BLAST identity are reported as median (interquartile range); **Equine infectious anemia virus* is contained in the reagents used to prepare genome libraries. Legend: N, number; bp, base pairs.

**S3 Table. Comparison of CSF viruses detected by PCR and Sequencing**

| **Virus** | **PCR in CSF** | **Sequencing in CSF** |
| --- | --- | --- |
| **Detectable CSF HIV RNA, n %** | 20 / 74 (27.0%) | 0 |
| **CSF HIV RNA, copies/mL**  20 – 21 – 22 – 23 – 26 – 33 – 34 – 39 – 43 – 46 – 49 – 54 – 56 – 57  – 58 – 72 – 77 – 88 – 118 – 133 | | - |
| **Detectable CSF EBV DNA, n %** | 3 / 72 (4.2%) | 1* |
| **CSF EBV DNA, copies/mL**  30 – 30 – 42 | | - |
| **Detectable CSF CMV DNA, n %** | 0 / 72 (0%) | 0 |
| **CSF CMV DNA, copies/mL**  None | | - |
| **Detectable CSF JCV DNA, n** | 2 / 56 (3.6%) | 0 |
| **CSF JCV DNA, copies/mL**  16 – 30 | | - |

Participants were selected among those enrolled in prospective studies on neuro-HIV; for research purposes, and not clinical indications, most of the participants had EBV, CMV and JCV DNA measured in their CSF through real time PCR. *This participant was none of the 3 participants with PCR-positive EBV DNA in the CSF. Legend: PWH, participants with HIV; CSF, cerebrospinal fluid; EBV, Epstein Barr virus; CMV, Cytomegalovirus; JCV, John Cunningham virus.

**S4 Table. Clinical characteristics of the 8 participants with CSF positive for eukaryotic viruses after contigs reconstruction**

| **ID** | **CSF virus** | **Age, years** | **Sex** | **CD4+ T cell count at LP, cells/µL (%)** | **CSF PCR*, cp/mL** | **CSF characteristics** | **Medical history** |
| --- | --- | --- | --- | --- | --- | --- | --- |
| **15**  **PWH** | **HPV** | 45-50 | MTF | 171 (11.5%), INR | 58 HIV RNA  Neg others | glucose 56 mg/dL  protein 54 mg/dL  0 cells/mL  iBBB – IS | Negative history for HPV-related lesions |
| **52**  **PWH** | **HPV**  **TBRFV**  **STV** | 50-55 | F | 50 (16.0%), INR | Neg | glucose 40 mg/dL  protein 36 mg/dL  0 cells/mL  nBBB – IS | Negative history for HPV-related lesions  Chronic diverticulosis, recurrent diverticulitis (rifaximin suppressive therapy) |
| **194**  **PWH** | **HHV6** | 40-45 | M | 413 (49.0%) | Neg | glucose 62 mg/dL  protein 48 mg/dL  0 cells/mL | Negative history for HHV6-related disorders |
| **214**  **PWH** | **EBV** | 45-50 | M | 357 (36.0%) | 57 HIV RNA  Neg others | glucose 62 mg/dL  protein 48 mg/dL  0 cells/mL  nBBB – NIS | Skin and gut HHV8-KS two years before and in remission at the LP (treated with ART and doxorubicin) |
| **404**  **PWH** | **HCV** | 60-65 | F | 965 (58.5%) | Neg | glucose 51 mg/dL  protein 43 mg/dL  0 cells/mL  nBBB – NIS | Untreated HCV infection (HCV RNA 1,263,977 UI/mL at the LP) |
| **4_14**  **CWH** | **GHV-GcV2** | 30-35 | M | - | - | glucose 57 mg/dL  protein 27 mg/dL  0 cells/mL  nBBB – NIS | SA for varicocelectomy  Negative medical history |
| **4_16**  **CWH** | **MCV** | 20-25 | M | - | - | glucose 62 mg/dL  protein 44 mg/dL  0 cells/mL  nBBB – NIS | SA for meniscus injury  Negative medical history |
| **4_19**  **CWH** | **HMCV** | 85-90 | M | - | - | glucose 65 mg/dL  protein 42 mg/dL  0 cells/mL  nBBB – NIS | SA for femur fracture  Medical history positive for benign prostate hyperplasia, cholecystectomy, hypertension, idiopathic pericarditis |

*RT-PCR for HIV RNA, EBV DNA, CMV DNA, and JCV DNA. Legend: ID, participant identification code; CSF, cerebrospinal fluid; MTF, male-to-female; INR, immunological non-responders (based on CD4+ T cell count and the duration of antiretroviral therapy: 23 months for ID15 and 49 months for ID52); i/nBBB, impaired/not impaired blood-brain barrier; (N)IS, (absence) presence of intrathecal synthesis; LP, lumbar puncture; TBRFV, *Tomato Brown Rugose Fruit virus*; STV, *Southern Tomato virus*; KS, Kaposi Sarcoma; ART, antiretroviral therapy; GHV-GcV2, *Gemycircularvirus HV-GcV2*; SA, Spinal anesthesia; MCV, *Molluscum Contagiosum virus*; HMCV, *Human Mastadenovirus type C*; NA, not available.

**S5 Table. Clinical characteristics of the 5 participants with CSF positive for prokaryotic viruses after contigs reconstruction**

| **ID** | **CSF virus** | **Age, years** | **Sex** | **CSF characteristics** | **Medical history** | **Primary human niches for the predated bacteria** |
| --- | --- | --- | --- | --- | --- | --- |
| **4_14**  **CWH** | ***Pseudomonas* phage**  ***Acinetobacter* phage** | 30-35 | M | glucose 57 mg/dL  protein 27 mg/dL  0 cells/mL  nBBB – NIS | SA for varicocelectomy  Negative medical history | *Pseudomonas* spp skin, airways, gut [1–3]  *Acinetobacter* spp  gut, airways, skin [4,5] |
| **40**  **PWH** | ***Burkholderia* phage JG068** | 35-40 | F | glucose 54 mg/dL  protein 40 mg/dL  0 cells/mL  nBBB – IS | CD4+ T cell 789/µL (48.0%)  COPD | *Burkholderia* spp airways (lung diseases [6,7]) |
| **157**  **PWH** | ***Cronobacter* phage** | 45-50 | F | glucose 55 mg/dL  protein 32 mg/dL  0 cells/mL  nBBB – NIS | CD4+ T cell 379/µL (28.0%)  HCV-related chronic hepatitis | *Cronobacter* spp gut (dry foods; reported neurotropism [8–10]) |
| **162**  **PWH** | ***Pseudomonas* phage** | 50-55 | F | glucose 56 mg/dL  protein 2 mg/dL  0 cells/mL  nBBB – NIS | CD4+ T cell 883/µL (33.0%)  Chronic low dose methylprednisolone and hydroxychloroquine for rheumatic arthritis  Chronic gastritis | *Pseudomonas* spp  Skin, airways, gut [1–3] |
| **175**  **PWH** | ***Corynebacterium***  **phage** | 40-45 | M | glucose 59 mg/dL  protein 41 mg/dL  0 cells/mL  nBBB – NIS | CD4+ T cell 164/µL (11.0%), INR (ART started 12 months before the LP)  Medical history negative but AIDS one year prior | *Corynebacterium* spp gut [11–13] |

Legend: ID, participant identification code; i/nBBB, impaired/not impaired blood-brain barrier; (N)IS, (absence) presence of intrathecal synthesis; CSF, cerebrospinal fluid; CWH, control without HIV infection; GHV-GcV2, *Gemycircularvirus HV-GcV2*; SA, Spinal anesthesia; PWH, participant with HIV infection; MSSA, Methicillin-susceptible Staphylococcus aureus; COPD, Chronic obstructive pulmonary disease.

**S1 Material. Case-series description of the 12 participants positive for CSF viruses after contigs reconstruction**

Among the five people with HIV (PWH) with hEV (see Figure 3 and S4 Table), the participant with CSF HCV (ID404) had been HCV-positive due to intravenous drug use 14 years prior to the lumbar puncture. At the time of CSF collection, HCV RNA levels in the blood were >1,000,000 IU/mL. Notably, CSAR was not indicative of blood-brain barrier (BBB) permeability. Neither participant ID15 nor ID52, who had CSF HPV, had a medical history of HPV-related lesions (e.g., verrucae, condyloma, cancer). Additionally, participant ID52 also had two tomato plant viruses in the CSF. Given the participant's medical history of chronic diverticulosis and recurrent diverticulitis, we hypothesized a gastrointestinal origin of these viruses through an impaired gut mucosa barrier; even this participant had a CSAR (4.1) not suggestive of BBB permeability.

Participant ID214 was positive for EBV in the CSF (with a genome coverage of 2.64%) but had negative CSF EBV DNA according to our in-house real-time PCR, and a negative history for EBV-related disorders (EBV serology was not available). This participant was diagnosed with skin and duodenum Kaposi's sarcoma two years before CSF collection, with remission achieved through ART and chemotherapy. The percentage of sequence homology between specific genes of HHV-8 and EBV ranges from 57% to 70% [14,15]. HHV-8 has been detected in the CSF and brain tissue of PWH [16,17], and the contigs identified in our participant were from EBV BPLF1 gene, which encodes a tegument protein conserved across the *Herpesviridae* family that is homologous to HHV-8 ORF64 [18]; fifty-four of 75 HHV-8 genes are collinear with their EBV homologs, and among these, the average amino acid identity is 35% [19]. Thus, we cannot rule out an incorrect taxonomic assignment of the contig, corresponding to <3% (30 bp) of the total sequence (~3000 bp), within the *Herpesviridae,* and specifically, to EBV instead of HHV-8.

Participant ID194 had the CSF positive for HHV-6, which latently infects >90% of the adult population worldwide [20]. The clinical history was negative for HHV-6 encephalitis and HHV6-associated disorders (e.g., leukemia, multiple sclerosis). Apart from HIV infection (under effective ART), the participant had no cause of immunodepression that may favor HHV-6 reactivation. However, while the frequency and intensity of HHV-6 reactivations in human saliva and blood has been described [21,22], this information for the CSF compartment is missing. Furthermore, sequencing genomic material cannot differentiate between active replication, latency, and chromosomally integration of HHV-6, which occur in ~1-2% of the general population [23]. Further clinical details for participants with CSF hEV are summarized in S4 Table.

Among the 3 participants without HIV infection (CWH) with hEV in their CSF, none suffered from any immunosuppressive condition nor BBB impairment. All were clinically asymptomatic except for the reasons to undergo surgery (see S4 Table). Of interest, participant ID4_19, with *Human Mastadenovirus C* in the CSF, had a single episode of idiopathic pericarditis in the past. *Mastadenovirus C* commonly causes upper and lower tract respiratory infections in pediatric populations [24] and can lead to encephalitis in immunocompromised patients [25], but it has also been implicated as a causative agent of pericarditis and myocarditis when NGS was used [26].

*Gemycircularvirus HV-GcV2*, found in the CSF of participant ID4_14, has recently been proposed as an etiological agent of various human infections, including of the CNS [27]. However, its pathogenicity remains unclear, as it has been also detected in the blood of asymptomatic individuals [28]. We included *Genomoviridae* reads among hEV, as most of the reads belonged to *Gemycircularvirus HV-GcV2*, whose recognized host are humans (taxonomy ID NCBI:txid1862825), and other detected species have also been reported as etiological agents of human infections [29]. However, this family has been detected also in other mammals, plants, and fungi [30], therefore, laboratory contamination or translocation from the gut cannot be ruled out. Future discoveries about this family may partially change their full classification as hEV. Lastly, *Molluscum contagiosum* is prevalent among young adults [31]. The silent medical history of participant ID4_16 (24 years old), recorded before CSF results, does not exclude the possibility of unreported symptomatic episodes or asymptomatic carriage. Of note, a previous case of mild self-resolutive encephalitis due to *Molluscum contagiosum virus* detected by NGS in the CSF of an otherwise healthy 25-year-old woman has been described [32].

Among the 5 CSF samples positive for 6 PV, all but one were from PWH (see S5 Table). None of the participants had a medical history indicating infections by the bacterial hosts of the identified CSF phages (e.g., *Acinetobacter baumannii*, *Pseudomonas aeruginosa*). It should be noted that our sequencing depth is limited in accurately attributing taxa at the species level. Different species of phages within the same family can infect various bacterial species within the same genus (e.g., *Pseudomonas*) [33]. Consequently, we cannot attribute the identified phages to a unique bacterial species, but to groups of predated bacteria, which include pathogenic, commensal and opportunistic species [34,35]. For instance, participant ID40 had *Burkholderia phage JG068* in the CSF but had no history of *Burkholderia* spp infections. However, the participant suffered from chronic obstructive pulmonary disease and recurrent pneumonias without available microbial isolates in the past, possibly indicating *Burkholderia* colonization.

**S6 Table.** **Results of CSF biomarkers and neurocognitive assessment**

| **Variable** | **N= 74 PWH** | **N=11 CWH** | **P** |
| --- | --- | --- | --- |
| **CSF leukocytes, cells/mL** | 0 (0-0) | 0 (0-0) | 0.412 |
| **CSF protein, mg/dL** | 43 (33-54) | 49 (32-56) | 0.575 |
| **CSF glucose, mg/dL** | 59 (54-64) | 61 (58-65) | 0.209 |
| **BBB impairment, n**  **CSAR** | 16 (21.6%)  5.3 (3.9-6.9) | 2 (18.2%)  6.5 (3.9-7.9) | 1.000  0.313 |
| **Intrathecal synthesis, n**  **IgG index** | 20 (27.0%)  0.33 (0.22-0.46) | 0 (0%)  0.36 (0.20-0.40) | 0.059  0.468 |
| **CSF total tau, pg/mL** | 134 (38-214) | - | - |
| **CSF 181-ptau, pg/mL** | 35 (27-45) | - | - |
| **CSF βA42, pg/mL** | 878 (656-1080) | - | - |
| **CSF neopterin, ng/mL** | 0.60 (0.34-1.09) | - | - |
| **CSF S100β, pg/mL** | 128 (83-186) | - | - |
| **GDS**  **Cognitive impairment ≥0.5** | 0.24 (0.09-0.66)  21/61 (34.4%) | - | - |
| **Memory domain score** | 0.3 (0.08-0.96) | - | - |
| **Attention/working memory domain score** | 0.1 (0.0-0.55) | - | - |
| **Language domain score** | 0.0 (0.0-0.35) | - | - |
| **Executive function domain score** | 0.0 (0.0-0.40) | - | - |
| **Motor function domain score** | 0.25 (0.0-1.00) | - | - |
| **BDI II, score**  **None/minimal <14**  **Mild 14-19**  **Moderate 20-28**  **Severe ≥29** | 8 (3-15)  47/66 (71.2%)  11/66 (16.7%)  4/66 (6.1%)  4/66 (6.1%) | - | - |

Legend: PWH, participants with HIV; CWH, control participants without HIV; CSF, cerebrospinal fluid; BBB, blood-brain barrier; CSAR, CSF-to-serum albumin ratio; GDS, Global Deficit Score; BDI II, Beck Depression Inventory II.

**S7 Table. Correlations between relative abundance of CSF viral categories and neurocognitive measures**

| **Cognitive scores** | **RA of hEV** | **RA of nhEV** | **RA of PV** |
| --- | --- | --- | --- |
|  | **Rho, p value (n=61)** | | |
| **GDS** | **-,376, 0.003** | **,256, 0.046** | ,230, 0.075 |
| **Memory** | **-,279, 0.030** | ,239, 0.064 | ,166, 0.201 |
| **Attention/Work. memory** | **-,353, 0.005** | -,039, 0.767 | -,070, 0.590 |
| **Language** | -,230, 0.074 | ,056, 0.666 | ,023, 0.858 |
| **Executive functions** | **-,299, 0.019** | **,292, 0.022** | ,223, 0.083 |
| **Motor functions** | -,244, 0.058 | ,190, 0.143 | ,170, 0.191 |
| **Depressive symptoms** | **Rho, p value (n=66)** | | |
| **BDI-II** | ,087, 0.485 | -,049, 0.698 | -,036, 0.774 |
| **Sensitivity analyses in positive CSF samples only** | | | |
| **Cognitive scores** | **Rho, p value (n=27)** | | |
| **GDS** | **-,654, <0.001** | ,325, 0.098 | ,349, 0.074 |
| **Memory** | **-,517, 0.006** | ,327, 0.096 | ,192, 0.182 |
| **Attention/Work. memory** | **-,386, 0.047** | ,225, 0.258 | ,182, 0.364 |
| **Language** | -,350, 0.074 | ,140, 0.485 | ,232, 0.244 |
| **Executive functions** | **-,616, <0.001** | ,358, 0.067 | ,319, 0.105 |
| **Motor functions** | **-,553, 0.003** | ,271, 0.171 | ,266, 0.180 |
| **Depressive symptoms** | **Rho, p value (n=32)** | | |
| **BDI-II** | ,138, 0.451 | -,146, 0.424 | -,090, 0.622 |

Legend: RA, relative abundance; hEV, human eukaryotic viruses; nhEV, non-human eukaryotic viruses; PV, prokaryotic viruses; GDS, Global Deficit Score; Work. Memory, working memory; BDI-II, Beck Depression Inventory II.

**S8 Table. Comparisons of Demographic, HIV-related parameters, Neuroinflammation and Neurocognitive measures between CSF Clusters**

| **Characteristic** | **C1**  **(N = 36)** | **C2**  **(N = 24)** | **C3**  **(N = 14)** | **P^1^** | **C1vsC2^1^** | **C1vsC3^1^** | **C2vsC3^1^** |
| --- | --- | --- | --- | --- | --- | --- | --- |
| **Age, years** | 51 (9) | 51 (13) | 45 (14) | 0.27 | 0.84 | 0.12 | 0.18 |
| **Female gender, n** | 7 (19%) | 14 (58%) | 3 (21%) | **0.004** | **0.002** | 0.87 | **0.027** |
| **Ethnicity, white, n** | 34 (94%) | 22 (92%) | 13 (93%) | 0.91 | 0.67 | 0.83 | 0.90 |
| **Education, years** | 10 (4) | 10 (4) | 10 (2) | 0.99 | 0.82 | 0.92 | 0.97 |
| **HIV transmission routes, n**  **MSM**  **Heterosexual**  **Past iv drug use** | 13 (36%)  10 (28%)  13 (36%) | 7 (29%)  10 (42%)  7 (29%) | 4 (29%)  6 (43%)  4 (29%) | 0.80 | 0.54 | 0.59 | 0.99 |
| **Detectable CSF HIV RNA, n** | 10 (28%) | 6 (25%) | 4 (29%) | 0.96 | 0.81 | 0.96 | 0.81 |
| **CSF HIV RNA, cp/mL** | 27 (18) | 27 (24) | 33 (30) | 0.90 | 0.77 | 0.81 | 0.67 |
| **Detectable plasma HIV RNA, n** | 9 (25%) | 2 (8.3%) | 2 (14%) | 0.24 | 0.10 | 0.41 | 0.56 |
| **Plasma HIV RNA, cp/mL** | 28 (22) | 24 (22) | 24 (15) | 0.27 | 0.12 | 0.44 | 0.57 |
| **CD4+ T cell count, cells/mmc** | 428 (254) | 617 (255) | 409 (217) | **0.009** | **0.010** | 0.71 | **0.008** |
| **CD4+ T cell count, %** | 25 (12) | 33 (11) | 28 (10) | **0.021** | **0.008** | 0.23 | 0.21 |
| **CD4/CD8 ratio** | 0.68 (0.43) | 1.04 (0.75) | 0.83 (0.45) | 0.050 | **0.026** | 0.26 | 0.43 |
| **CD4+ T cell count nadir, cells/mmc** | 178 (141) | 173 (151) | 147 (119) | 0.82 | 0.85 | 0.45 | 0.87 |
| **Past AIDS, n** | 23 (64%) | 15 (63%) | 8 (57%) | 0.91 | 0.91 | 0.66 | 0.74 |
| **ART regimen, n**  **NNRTI-2NRTIs**  **PI-2NRTIs**  **INSTI-2NRTIs** | 7 (19%)  14 (39%)  15 (42%) | 11 (46%)  7 (29%)  6 (25%) | 2 (14%)  10 (71%)  2 (14%) | **0.021** | 0.087 | 0.10 | **0.037** |
| **Tourtelotte index** | 7 (16) | 5 (8) | 4 (5) | 0.85 | 0.75 | 0.53 | 0.96 |
| **Tibbling index** | 0.64 (0.36) | 0.70 (0.34) | 0.70 (0.28) | 0.39 | 0.31 | 0.23 | 0.74 |
| **IgG index** | 0.44 (0.33) | 0.36 (0.18) | 0.33 (0.14) | 0.89 | 0.78 | 0.70 | 0.69 |
| **CSF-to-serum Albumin ratio** | 6.09 (2.89) | 5.57 (2.63) | 4.87 (1.57) | 0.36 | 0.36 | 0.19 | 0.55 |
| **Permeable BBB, n** | 10 (28%) | 5 (21%) | 1 (7%) | 0.28 | 0.54 | 0.11 | 0.26 |
| **CSF total tau protein, pg/mL** | 155 (87) | 185 (148) | 98 (73) | 0.12 | 0.76 | **0.030** | 0.13 |
| **CSF 181-ptau protein, pg/mL** | 38 (16) | 42 (18) | 33 (12) | 0.35 | 0.33 | 0.43 | 0.18 |
| **CSF beta amyloid 1-42, pg/mL** | 901 (313) | 1,029 (468) | 737 (185) | **0.042** | 0.59 | **0.032** | **0.031** |
| **CSF S100beta protein, pg/mL** | 130 (97) | 225 (263) | 138 (50) | **0.025** | **0.016** | 0.17 | 0.38 |
| **CSF neopterin, ng/mL** | 0.83 (0.56) | 0.98 (0.98) | 0.65 (0.61) | 0.47 | 0.94 | 0.21 | 0.37 |
| **CSF leukocytes, cells/mL** | 1 (7) | 3 (12) | 0 (0) | 0.40 | 0.12 | 0.20 | **0.036** |
| **CSF protein, mg/dL** | 48 (18) | 49 (23) | 40 (11) | 0.42 | 0.59 | 0.15 | 0.65 |
| **CSF glucose, mg/dL** | 61 (11) | 60 (13) | 58 (12) | 0.58 | 0.40 | 0.69 | 0.38 |
|  | **N=34** | **N=18** | **N=9** |  |  |  |  |
| **GDS** | 0.52 (0.82) | 0.69 (0.72) | 0.16 (0.17) | **0.020** | 0.080 | **0.022** | **0.013** |
| **Cognitive impairment (GDS >0.5), n** | 11 (32%) | 10 (56%) | 0 (0%) | **0.015** | 0.10 | **0.048** | **0.005** |
| **Memory domain score** | 0.62 (0.82) | 0.95 (0.94) | 0.39 (0.62) | 0.23 | 0.21 | 0.37 | 0.13 |
| **Attention domain score** | 0.56 (0.79) | 0.36 (0.65) | 0.09 (0.23) | 0.053 | 0.22 | **0.004** | 0.18 |
| **Language domain score** | 0.45 (0.92) | 0.38 (0.83) | 0.07 (0.17) | 0.30 | 0.92 | **0.024** | 0.14 |
| **Executive functions domain score** | 0.38 (0.89) | 0.71 (1.04) | 0.04 (0.13) | **0.015** | **0.045** | **0.032** | **0.009** |
| **Motor function domain score** | 0.61 (1.10) | 1.04 (1.11) | 0.20 (0.31) | **0.047** | 0.073 | 0.30 | **0.018** |
|  | **N=34** | **N=20** | **N=12** |  |  |  |  |
| **Beck Depression Inventory II score** | 10 (10) | 10 (8) | 11 (9) | 0.81 | 0.80 | 0.55 | 0.61 |
| **Depressive mood (BDI II≥14), n** | 9 (26%) | 5 (25%) | 5 (42%) | 0.55 | 0.91 | 0.32 | 0.32 |

^1^Fisher’s exact test, Kruskal-Wallis test, ANOVA, Chi-squared test, according to type and distribution of the variable. Legend: C, cluster; MSM, males who have sex with other males; pIDU, past intravenous drug users; CSF, cerebrospinal fluid; ART, antiretroviral therapy; PI, protease inhibitors; NNRTI, non-nucleoside reverse transcriptase inhibitors; INSTI, integrase strand-transfer inhibitors; BBB, blood-brain barrier; 181-p-tau, 181-phosphorylated tau protein; AB42, fragment 1-42 of beta amyloid; GDS, Global Deficit Score; BDI-II, Beck Depression Inventory II.

**S9 Table. Multivariate analysis for neurocognitive metrics (n=61)**

| **Outcome** | **Independent variable** | **aβ* (95% CI)** | **p** |
| --- | --- | --- | --- |
| **GDS** | **C1 vs C3**  **C2 vs C3**  **C1 vs C2** | 0.07 (-0.035; 0.19)  **0.15 (0.022; 0.28)**  -0.075 (-0.17; 0.021) | 0.179  **0.021**  0.126 |
| **Memory deficit score** | **C1 vs C3**  **C2 vs C3**  **C1 vs C2** | 0.046 (-0.093; 0.19)  0.13 (-0.034; 0.29)  -0.082 (-0.20; 0.039) | 0.524  0.123  0.184 |
| **Attention/working memory deficit score** | **C1 vs C3**  **C2 vs C3**  **C1 vs C2** | **0.12 (0.00; 0.24)**  0.11 (-0.035; 0.25)  0.014 (-0.092; 0.12) | **0.050**  0.140  0.790 |
| **Language deficit score** | **C1 vs C3**  **C2 vs C3**  **C1 vs C2** | 0.089 (-0.038; 0.22)  0.097 (-0.051; 0.24)  -0.0079 (-0.12; 0.10) | 0.169  0.200  0.890 |
| **Executive function deficit score** | **C1 vs C3**  **C2 vs C3**  **C1 vs C2** | 0.064 (-0.056; 0.18)  **0.16 (0.019; 0.30)**  -0.095 (-0.200; 0.010) | 0.293  **0.026**  0.077 |
| **Motor function deficit score** | **C1 vs C3**  **C2 vs C3**  **C1 vs C2** | 0.059 (-0.087; 0.20)  **0.21 (0.033; 0.37)**  **-0.14 (-0.27; -0.016)** | 0.427  **0.019**  **0.027** |

*Each generalized linear model included cluster membership as independent variable, and was adjusted by age, sex, CD4+ T cell count, and ART regimens.

**S10 Table. Battery of cognitive tests used for neurocognitive assessment**

| **Cognitive domain** | **Tests** |
| --- | --- |
| **Attention/Working Memory** | - Trail Making test Part A and B - Stroop Color test - Digit Symbol test |
| **Short- and long-term Memory** | - Delayed recall of the Rey-Osterrieth complex figure - Disyllabic words serial repetition test - Corsi block-tapping test - Prose memory story - Digit span forward test |
| **Language** | - Semantic verbal fluency - Phonemic verbal fluency |
| **Executive Functions** | - Frontal Assessment Battery |
| **Motor Functions** | - Groove Pegboard test for the dominant and non-dominant hand |

**S11 Table. Missing data**

| **Variables with missing data** | **N of missing values** | |
| --- | --- | --- |
|  | **PWH (n=74)** | **CWH (n=11)** |
| **CD4+ T cell nadir** | 4 (5.4%) | NP |
| **IgG index** | 3 (4.1%) | 0 |
| **CSF total tau** | 4 (5.4%) | NP |
| **CSF 181-p-tau** | 3 (4.1%) | NP |
| **CSF beta amyloid 1-42** | 3 (4.1%) | NP |
| **CSF S100 beta protein** | 4 (5.4%) | NP |
| **CSF neopterin** | 2 (2.7%) | NP |
| **CSF leukocytes** | 2 (2.7%) | 0 |
| **CSF proteins** | 2 (2.7%) | 0 |
| **CSF glucose** | 2 (2.7%) | 0 |
| **Cognitive assessment** | 13 (17.5%) | NP |
| **Depressive mood assessment** | 8 (10.8%) | NP |

Legend: PWH, participants with HIV; CWH, participants without HIV; N, number; NP, analysis not performed in CWH; CSAR, CSF-to-Serum albumin ratio; CSF, cerebrospinal fluid; 181-p-tau, 181-phosphorylated tau.

**Supplementary References (S5 Table and S1 Material):**

1. Martak D, Gbaguidi-Haore H, Meunier A, et al. High prevalence of Pseudomonas aeruginosa carriage in residents of French and German long-term care facilities. Clin Microbiol Infect **2022**; 28:1353–1358.

2. Okuda J, Hayashi N, Okamoto M, et al. Translocation of Pseudomonas aeruginosa from the Intestinal Tract Is Mediated by the Binding of ExoS to an Na,K-ATPase Regulator, FXYD3. Infection and Immunity **2010**; 78:4511–4522.

3. Wheatley RM, Caballero JD, van der Schalk TE, et al. Gut to lung translocation and antibiotic mediated selection shape the dynamics of Pseudomonas aeruginosa in an ICU patient. Nat Commun **2022**; 13:6523.

4. Glover JS, Browning BD, Ticer TD, Engevik AC, Engevik MA. Acinetobacter calcoaceticus is Well Adapted to Withstand Intestinal Stressors and Modulate the Gut Epithelium. Front Physiol **2022**; 13:880024.

5. Ketter PM, Yu J-J, Guentzel MN, et al. Acinetobacter baumannii Gastrointestinal Colonization Is Facilitated by Secretory IgA Which Is Reductively Dissociated by Bacterial Thioredoxin A. mBio **2018**; 9:e01298-18.

6. Mukhopadhyay C, Bhargava A, Ayyagari A. Two Novel Clinical Presentations of Burkholderia cepacia Infection. J Clin Microbiol **2004**; 42:3904–3905.

7. Lynch KH, Abdu AH, Schobert M, Dennis JJ. Genomic characterization of JG068, a novel virulent podovirus active against Burkholderia cenocepacia. BMC Genomics **2013**; 14:574.

8. Blackwood BP, Hunter CJ. Cronobacter spp. Microbiol Spectr **2016**; 4.

9. Yan Q q., Condell O, Power K, Butler F, Tall B d., Fanning S. Cronobacter species (formerly known as Enterobacter sakazakii) in powdered infant formula: a review of our current understanding of the biology of this bacterium. Journal of Applied Microbiology **2012**; 113:1–15.

10. Gan X, Li M, Yan S, Wang X, Wang W, Li F. Genomic Landscape and Phenotypic Assessment of Cronobacter sakazakii Isolated From Raw Material, Environment, and Production Facilities in Powdered Infant Formula Factories in China. Front Microbiol **2021**; 12:686189.

11. Rajilić-Stojanović M, de Vos WM. The first 1000 cultured species of the human gastrointestinal microbiota. FEMS Microbiology Reviews **2014**; 38:996–1047.

12. Ye J, Li Y, Wang X, et al. Positive interactions among Corynebacterium glutamicum and keystone bacteria producing SCFAs benefited T2D mice to rebuild gut eubiosis. Food Res Int **2023**; 172:113163.

13. Sędzikowska A, Szablewski L. Human Gut Microbiota in Health and Selected Cancers. Int J Mol Sci **2021**; 22:13440.

14. Russo JJ, Bohenzky RA, Chien M-C, et al. Nucleotide sequence of the Kaposi sarcoma-associated herpesvirus (HHV8). Proceedings of the National Academy of Sciences **1996**; 93:14862–14867.

15. AuCoin DP, Colletti KS, Cei SA, Papousková I, Tarrant M, Pari GS. Amplification of the Kaposi’s sarcoma-associated herpesvirus/human herpesvirus 8 lytic origin of DNA replication is dependent upon a *cis*-acting AT-rich region and an ORF50 response element and the *trans*-acting factors ORF50 (K-Rta) and K8 (K-bZIP). Virology **2004**; 318:542–555.

16. Broccolo F, Iuliano R, Careddu AM, et al. Detection of lymphotropic herpesvirus DNA by polymerase chain reaction in cerebrospinal fluid of AIDS patients with neurological disease. Acta Virol **2000**; 44:137–143.

17. Tso FY, Sawyer A, Kwon EH, et al. Kaposi’s Sarcoma–Associated Herpesvirus Infection of Neurons in HIV-Positive Patients. J Infect Dis **2017**; 215:1898–1907.

18. Murata T. Tegument proteins of Epstein-Barr virus: Diverse functions, complex networks, and oncogenesis. Tumour Virus Research **2023**; 15:200260.

19. Edelman DC. Human herpesvirus 8 – A novel human pathogen. Virology Journal **2005**; 2:78.

20. Emery VC, Clark DA. HHV-6A, 6B, and 7: persistence in the population, epidemiology and transmission. In: Arvin A, Campadelli-Fiume G, Mocarski E, et al., eds. Human Herpesviruses: Biology, Therapy, and Immunoprophylaxis. Cambridge: Cambridge University Press, 2007. Available at: http://www.ncbi.nlm.nih.gov/books/NBK47441/. Accessed 29 February 2024.

21. Agut H, Bonnafous P, Gautheret-Dejean A. Laboratory and Clinical Aspects of Human Herpesvirus 6 Infections. Clin Microbiol Rev **2015**; 28:313–335.

22. Harnett GB, Farr TJ, Pietroboni GR, Bucens MR. Frequent shedding of human herpesvirus 6 in saliva. J Med Virol **1990**; 30:128–130.

23. Pellett PE, Ablashi DV, Ambros PF, et al. Chromosomally integrated human herpesvirus 6: questions and answers. Rev Med Virol **2012**; 22:144–155.

24. Takahashi K, Gonzalez G, Kobayashi M, et al. Pediatric Infections by Human mastadenovirus C Types 2, 89, and a Recombinant Type Detected in Japan between 2011 and 2018. Viruses **2019**; 11:1131.

25. Tunkel AR, Baron EL, Buch KA, Marty FM, Martinez-Lage M. Case 31-2019: A 45-Year-Old Woman with Headache and Somnolence. N Engl J Med **2019**; 381:1459–1470.

26. Lin L, Xu M, Zhang H. Assistance of metagenomics next-generation sequencing for diagnosis of adenovirus pericarditis with pericardial effusion in a child: a case report and literature review. Front Pediatr **2023**; 11:1174326.

27. Phan TG, Mori D, Deng X, et al. Small circular single stranded DNA viral genomes in unexplained cases of human encephalitis, diarrhea, and in untreated sewage. Virology **2015**; 482:98–104.

28. Breno Zampieri Lima M, Giovana Pereira Daniel T, Tayaná Oliveira Bitencourt H, et al. Molecular frequency of human gemycircularvirus (GCYV) dna among blood donors from the Brazilian Amazon. Transfus Clin Biol **2024**; :S1246-7820(24)00035–1.

29. Wang J, Li Y, He X, et al. Gemykibivirus Genome in Lower Respiratory Tract of Elderly Woman With Unexplained Acute Respiratory Distress Syndrome. Clinical Infectious Diseases **2019**; 69:861–864.

30. Varsani A, Krupovic M. Family Genomoviridae: 2021 taxonomy update. Arch Virol **2021**; 166:2911–2926.

31. Hebert AA, Bhatia N, Del Rosso JQ. Molluscum Contagiosum: Epidemiology, Considerations, Treatment Options, and Therapeutic Gaps. J Clin Aesthet Dermatol **2023**; 16:S4–S11.

32. Huang M, Peng M, Gan CH, et al. A case of intracranial molluscum contagiosum virus infection diagnosed by metagenomic sequencing of cerebrospinal fluid. Acta Virol **2019**; 63:333–337.

33. Campbell RA, Farlow J, Freyberger HR, et al. Genome Sequences of 17 Diverse Pseudomonas aeruginosa Phages. Microbiology Resource Announcements **2021**; 10:10.1128/mra.00031-21.

34. Feng D-Y, Zhou J-X, Li X, Wu W-B, Zhou Y-Q, Zhang T-T. Differentiation Between Acinetobacter Baumannii Colonization and Infection and the Clinical Outcome Prediction by Infection in Lower Respiratory Tract. Infect Drug Resist **2022**; 15:5401–5409.

35. Coutinho CP, dos Santos SC, Madeira A, Mira NP, Moreira AS, Sá-Correia I. Long-Term Colonization of the Cystic Fibrosis Lung by Burkholderia cepacia Complex Bacteria: Epidemiology, Clonal Variation, and Genome-Wide Expression Alterations. Front Cell Infect Microbiol **2011**; 1:12.
